# Antimicrobial peptide plectasin recombinantly produced in *Escherichia coli* disintegrates cell walls of gram-positive bacteria, as proven by transmission electron and atomic force microscopy

**DOI:** 10.1128/jb.00456-24

**Published:** 2025-04-04

**Authors:** Matthias Müller, Sigrid Mayrhofer, Wisnu Arfian A. Sudjarwo, Martin Gibisch, Christopher Tauer, Eva Berger, Cécile Brocard, José L. Toca-Herrera, Gerald Striedner, Rainer Hahn, Monika Cserjan-Puschmann

**Affiliations:** 1 Christian Doppler Laboratory for production of next-level biopharmaceuticals in E. coli, Institute of Bioprocess Science and Engineering, BOKU University 27270, Vienna, Austria; 2 Institute of Molecular Biotechnology, BOKU University 686422 , Vienna, Austria; 3 Institute of Biophysics, BOKU University 27270, Vienna, Austria; 4 Boehringer Ingelheim RCV GmbH & Co KG, Dr. Boehringer-Gasse 33433 , Vienna, Austria; University of Southern California, Los Angeles, California, USA

**Keywords:** recombinant peptides, *E. coli*, CASPON-tag, purification, disulfide bridges

## Abstract

Plectasin, an antimicrobial peptide, was initially isolated from the saprophytic fungus *Pseudoplectania nigrella*. This peptide, a member of the cysteine-stabilized α-helix and β-sheet family, has demonstrated potent antimicrobial activity against gram-positive pathogens, including strains resistant to conventional antibiotics. Our CASPON platform process enables the production of substantial quantities of plectasin, facilitating investigations on the activity and the mode of action of this recombinantly produced peptide. To this end, we developed an activity assay that reflects the growth inhibition of selected model bacteria, allowing for statistical analysis and evaluation of reproducibility. The mode of action was investigated using transmission electron microscopy and atomic force microscopy. The latter provided new insights into alterations in the cell surface of gram-positive bacteria treated with plectasin at the single-cell level. While the cell diameter remained unaltered, the roughness increased by up to twofold, and the cell stiffness decreased by approximately one-third in the four gram-positive bacterial strains tested. Statistical analysis of these morphological changes provides further insights into the effects and efficiency of antimicrobial peptides targeting pathogen cell walls.

**IMPORTANCE:**

The rise of antibiotic-resistant bacteria is a major threat to global health. Antimicrobial peptides (AMPs) offer a promising way to combat this. With the CASPON technology, we produced the AMP plectasin comprising three disulfide bonds using *Escherichia coli*. The activity of purified plectasin with and without a CASPON fusion tag was determined for four gram-positive and four gram-negative bacteria. As anticipated, only gram-positive bacteria showed a growth inhibition response to un-tagged plectasin. Plectasin treatment on gram-positive bacteria was visualized via electron microscopy. Evaluation of atomic force microscopy indicated that plectasin treatment led to increased roughness but maintained thickness. Based on our study, we assume that the CASPON technology can be employed in the future for the production and characterization of medical-grade AMPs.

## INTRODUCTION

The extensive use of antimicrobials in humans, livestock, and agriculture has resulted in the emergence of numerous drug-resistant strains (1–3). This trend poses a critical threat in the future, making the development of antibiotic alternatives a pressing and ongoing research focus (4, 5).

Antimicrobial peptides (AMPs), also known as "defensins", are promising alternatives to conventional antibiotics. Produced by a multitude of organisms, including bacteria, fungi, insects, plants, and vertebrates (6), these peptides are typically 33–44 amino acids long and comprise up to three disulfide bridges for their structural stability. They can inhibit bacterial growth, disable viruses, and even transform cancerous cells (7, 8). In comparison to conventional antibiotics, AMPs exhibit a broader treatment spectrum, highly selective toxicity, and a faster killing action (9, 10). Their classical mode of action involves damaging the bacterial cell membrane and/or the peptidoglycan layer (11).

Plectasin is an example of an agent that disrupts the ability of bacterial cells to maintain their peptidoglycan layer. Numerous studies have explored its potential to serve as an alternative treatment for multiresistant bacteria (12–15), although its precise mechanism remains unclear. Plectasin, the first defensin identified from a fungus, was discovered by extracting mRNA from the mycelia of *Pseudoplectania nigrella*, followed by cDNA synthesis and insertional mutagenesis (16). Its efficacious antimicrobial activities against gram-positive bacteria have been documented in numerous studies (13, 14, 16–18). Most studies indicate that plectasin interacts with the biosynthesis of the peptidoglycan layer by forming plectasin/lipid II complexes, preventing correct layer assembly (19–22). The peptide comprises 40 amino acids and contains three disulfide bonds essential for its biological function (12). Our recent studies, beginning with the production of peptides by *E. coli* in C-limited fed-batch cultivations (23) and continuing with the development of a downstream process for somatostatin-28 (SST) (24), have demonstrated that the required disulfide bonds of plectasin can be correctly formed and preserved during purification. The CASPON platform process is designed and optimized for efficient peptide and protein purification (25). Central to this platform is the CASPON-tag (26), a small (4.1 kDa) N-terminal tag that enhances the soluble expression of the protein/peptide of interest (POI) in *E. coli* through the T7AC sequence (27) and facilitates affinity chromatography via a 6-His sequence. It includes a recognition site for efficient tag removal by cpCasp2 (28) or the CASPON enzyme (29), generating an authentic N-terminus for the POI. This stabilizing tag facilitates effective peptide extraction and selective binding to purification resins. The CASPON purification process comprises four principal stages: acid extraction to release the target peptide from the periplasm of the host cell; immobilized metal affinity chromatography (IMAC) capture to bind the CASPON-tagged peptide to a metal-chelated resin, separating it from other impurities; CASPON enzyme-mediated tag cleavage to yield the native peptide; and CASPON-tag removal through subtractive IMAC and ion exchange chromatography to ensure final product purity. One limitation of this pathway is the high number of purification steps, which resulted in a relatively low total yield. To address this, we propose terminating the purification process after the IMAC capture step. Our findings demonstrate minimal impurity levels of host cell proteins (HCPs), DNA, and endotoxins solely with an acid extraction followed by a capture step (24).

It remains unclear whether biomolecules produced with and without CASPON-tag retain their activity once purified, as this has not been investigated in all CASPON-related studies (25, 29–32). Therefore, we developed an assay in this study to compare the antimicrobial activity of plectasin and CASPON-tagged plectasin, determining the minimum inhibitory concentration (MIC) of four gram-positive and four gram-negative bacterial strains. The strains, detailed in the *Materials and Methods* section, were selected based on EUCAST recommendations (33) and are commonly used in pathogen quality control. The outcome will indicate whether CASPON-tagged plectasin retains the same activity as the wild-type plectasin, potentially allowing for a shorter purification process and increased total yield. Alternatively, if the CASPON-tag impairs or eliminates the activity of plectasin, it may protect the bacterial cells from the recombinant product.

This would also enhance the understanding of how AMPs harm potential pathogens (34–36). Many pathogens that develop resistance to antibiotic agents are gram-positive (37, 38). The morphology and viability of these microorganisms depend on the proper formation of the peptidoglycan layer (39–41). AMPs (e.g., plectasin) have the capacity to disrupt the bacterial cell’s ability to maintain this layer (19, 20). Most studies on AMPs have employed microscopic analysis methods such as scanning electron microscopy (SEM) or transmission electron microscopy (TEM) (42–45). Nevertheless, alterations in the physical properties (e.g., shape, mechanics, and cell wall structure) of treated bacteria have not been observed.

Atomic force microscopy (AFM) is a microscopic method (46) recently used to measure bacterial mechanics, including stiffness, thickness, and roughness (47–51). Additionally, current studies have characterized gram-negative bacteria and their outer membranes after antibiotic treatment (52–56), providing crucial insights into the effectiveness of different antibiotics on various bacterial strains. Nevertheless, the impact of AMPs on target pathogens and the physical properties of gram-positive cells after treatment with agents that disrupt murein have not been well studied.

This study demonstrates the potential of the CASPON platform technology for the economical production and purification of AMPs, using plectasin as an example. By providing quantitative yields, purity analyses, and the development of an activity assay, the impact of the CASPON-tag on AMP functionality was evaluated, specifically whether it reduces or eliminates the antimicrobial activity. Furthermore, AFM is introduced as a sophisticated method to assess bacterial cell wall damage caused by AMPs. The mechanical properties and surface images of four gram-positive strains were analyzed using AFM, with the findings supported using TEM. The outcomes of this study provide a foundation for using the CASPON technology to produce AMPs with low impurities and high yields, and they highlight the potential of AFM to study the activity mechanisms of both established and as-yet-undiscovered AMPs.

## MATERIALS AND METHODS

### *E. coli* production strain and biomass

The peptide plectasin was expressed in the periplasm of *Escherichia coli* BL21(DE3) (New England Biolabs, USA). The peptide was fused to the N-terminal CASPON-tag (4.14 kDa) (32). For translocation, plectasin was expressed in combination with a signal sequence derived from outer membrane protein A (OmpA). Expression cassettes were designed and integrated into the genome according to our last study (24). The amino acid sequences of all signal sequences and peptides are listed in the supplementary material (Table S1). Further detailed information and a description of the fermentation process can be found in (23). After fermentation, the cell suspension was centrifuged at 18,000 *g* for 30 minutes with the batch centrifuge Sorvall Lynx 6000 (Thermo Fischer Scientific, Waltham, Massachusetts, USA). The biomass pellet was then taken and stored at −20°C for upcoming investigations.

### Purification steps

The purification was done in bench-scale to receive enough purified plectasin for needed experiments. The purification process was performed three times to prove the reproducibility.

#### Cell disintegration and clarification

The stored *E. coli* cell pellet was solubilized using extraction buffer (50 mM Tris, 300 mM NaCl, and pH 8.5). After 1 hour of stirring, the HCl solution (25%) was added to the suspension, creating a final CDM of 30 g/L and an HCl concentration of 2% (vol/vol). The suspension was incubated at room temperature for 3 hours and a stirring rate of 250 rpm. After incubation, the solution was centrifuged (Sorvall Lynx 6000, Thermo Fischer Scientific, Massachusetts, USA) at 18,000 *g* for 30 minutes. The supernatant was taken, and an imidazole solution (8 M) was added to reach a final concentration of 15 mM, avoiding impurity capture on the IMAC column.

#### IMAC capture

Chromatography capture runs were performed on an Äkta Pure system in combination with an S9 sample pump (Cytiva, Marlborough, Massachusetts, USA). The plectasin fused to the CASPON-tag was captured on an 8-mL Ni Sepharose 6 FF column (Tricorn 10, Cytiva), which was packed by flow packing in an 8-mL column. Buffer compositions and method are clearly described by Müller *et al*. (24).

#### Diafiltration and dilution

IMAC elution buffer was exchanged five times against PBS (137 mM NaCl, 17.8 Na_2_HPO_4_ × 2H_2_O, 13.6 mM KH_2_PO_4_, 2.7 mM KCl, and pH 7.4) with nominal 3 kDa cut-off in Amicon Ultra spin vials (Merck, Darmstadt, Germany). The buffer was exchanged discontinuously until the imidazole concentration was below 10 mM. To prevent loss in the form of precipitated plectasin, the CASPON-plectasin was diluted with PBS to a final concentration of 3.2 mg/mL.

#### Cleavage with the CASPON enzyme

The buffer exchanged solution was treated by digesting CASPON-plectasin, using a 1:100 (mol/mol) dilution of the CASPON enzyme per peptide at 37°C for 12 hours. To prevent caspase oxidation and to facilitate the cleavage process, dithiothreitol (DTT) was added to a final concentration of 1 mM DTT. The temperature was maintained by using an Eppendorf Thermoblock (Hamburg, Germany). The CASPON enzyme is a modification of T7AC-6H cpCasp2, which is described by Cserjan-Puschmann *et al*. (28).

#### Subtractive IMAC and dilution

The CASPON-tag removal pools were loaded on the subtractive IMAC, using the same column as in the first chromatography step. Buffer compositions and method are clearly described by Müller *et al*. (24). The product pool was diluted 1:2 with HQ-water to reduce the conductivity to 8 mS/cm. Additionally, the pH was reduced from 7.4 to 6.0 by adding 0.1 M HCl to make the next CEX step feasible (calculated pI of plectasin is 7.77).

#### Cation exchange—CEX

The diluted pool was captured on a 1-mL self-packed Capto S ImpAct column, which was equilibrated with 0.5 x PBS buffer (69 mM NaCl, 9 Na_2_HPO_4_ × 2H_2_O, 7 mM KH_2_PO_4_, 1.4 mM KCl, and pH 6.0). After capturing of plectasin, a linear gradient (40 CV) was applied against high salt buffer (1069 mM NaCl, 9 Na_2_HPO_4_ × 2H_2_O, 7 mM KH_2_PO_4_, 1.4 mM KCl, and pH 6.0). All steps were performed with a residence time of 4 minutes. The main peak during elution was collected and used for analytic measurements. After elution, the column was treated with 0.5 M NaOH, ensuring a full CIPing process.

#### Ultrafiltration

The pooled elution fraction of CEX was ultrafiltrated to the required plectasin concentration for activity assay, TEM, and AFM. For this concentration step, Amicon Ultra spin vials (Merck, Darmstadt, Germany) with a nominal 3 kDa cut-off were used.

### Antimicrobial activity assay

#### Bacterial strains for activity assay

The test panel included four gram-positive and four gram-negative bacterial strains, which were selected based on EUCAST recommendations and are commonly used in quality control for pathogen testing. Specifically, the test panel comprised the following four gram-positive strains: *Staphylococcus aureus* DSM 2569 (ATCC 29213), *Streptococcus pneumoniae* DSM 24048 (ATCC 49619), *Listeria monocytogenes* DSM 20600 (ATCC 15313), and *Enterococcus faecalis* DSM 2570 (ATCC 29212). The gram-negative strains included the following: *Pseudomonas aeruginosa* DSM 1117 (ATCC 27853), *Escherichia coli* DSM 1103 (ATCC 25922), *Escherichia coli* BL21(DE3) DSM 102052 (ATCC BAA-1025-B2), and *Escherichia coli* HMS174 DSM 5932 (ATCC 47011). These strains were obtained from the German Collection of Microorganisms and Cell Cultures GmbH (DSMZ, Leibnitz Institute, Braunschweig, Germany). The strains were maintained in cryo-stocks containing 20% glycerol at a temperature of −80°C. When needed, all gram-negative strains were streaked on MH-agar (Oxoid, Hampshire, UK) and all gram-positive strains on TSYE-agar, with the exception of the *Listeria monocytogenes* strain, for which BHI-agar was used. All strains were incubated overnight at 35°C.

#### Preparation of test plates

The plates were made fresh on the day of the assay by first adding 50 µL of sterile water (for the gram-positive strains) or PBS buffer pH 7.4 (for the gram-negative strains) into the wells of the first to 11th column of a polystyrene 96-well plate (Greiner, 655161, Kremsmünster, Austria). An ampicillin stock solution (6,400 µg/mL) was prepared and diluted in sterile water to obtain a final ampicillin solution of 64 µg/mL. Purified plectasin, 100 µL of ampicillin solution, CASPON-plectasin, and sterile water for the negative control were each dispensed into two wells of the 12th microtiter plate column and diluted twofold from the 11th to the second column by transferring 50 µL per well of the previous column into the next one (in the end, 50 µL of the 2nd was discarded) to obtain the ranges of 0.064–64 µg/mL for ampicillin, 32–32,768 µg/mL for CASPON-plectasin, and 0.5–512 µg/mL (*Streptococcus pneumoniae* and *Staphylococcus aureus*), 1–1,024 µg/mL (*Enterococcus faecalis* and *Listeria monocytogenes*), or 4–4,096 µg/mL (gram-negative strains) for plectasin.

#### Susceptibility testing

Bacterial inocula were prepared by suspending colonies from incubated plates described above with 3 mL 0.85% NaCl solution to obtain a turbidity corresponding to McFarland standard 0.5. The inoculated saline suspensions (1–2 × 10^8^ CFU/mL) were diluted 1:100 in double concentrated MH- (all gram-negative strains, *Staphylococcus aureus*, and *Enterococcus faecium*) or MHF- (*Streptococcus pneumoniae*, *Listeria monocytogenes*; MH-broth with 5% lysed horse blood and 20 mg/L ß-NAD) broth to obtain approximately 1–2 × 10^5^ CFU/mL. Fifty microliters of the diluted inoculum was added to the wells of the prepared microdilution plate, resulting in the final plectasin (0.25–256 µg/mL, 0.5–512 µg/mL, and 2–2,048 µg/mL), CASPON-plectasin (16–16,384 µg/mL), and ampicillin (0.032–32 µg/mL) concentrations and approximately 5 × 10^4^ CFU/well. The wells of the negative control were inoculated with sterile double-concentrated MH- or MH-F broth. A schematic illustration of the plate preparation can be found in the supplementary material (Fig. S1). Plates were incubated in ambient air at 35°C for 18 hours. After incubation, the plates were shaken for 1 minute (1,000 rpm) on a platform shaker (Titramax 101, Heidolph, Schwabach, Germany) until obtaining a homogeneous suspension. The absorbances (625 nm) of each well were measured on a Tecan Spark device (Männedorf, Switzerland) and subtracted by the average absorbance of the negative control. Differences around 0 indicated that no growth has occurred. The MIC values were determined as the lowest concentration at which growth was inhibited after 18 hours of incubation. The variability of the MIC results was evaluated by testing all strains against each agent in three independent assays. The accuracy of MIC testing was monitored by using ampicillin and comparing obtained MIC values to the acceptable MIC ranges of the tested quality control strains *Staphylococcus aureus* DSM 2569 (ATCC 29213), *Streptococcus pneumoniae* DSM 24048 (ATCC 49619), *Listeria monocytogenes* DSM 20600 (ATCC 15313), *Enterococcus faecalis* DSM 2570 (ATCC 29212), *Pseudomonas aeruginosa* DSM 1117 (ATCC 27853), and *Escherichia coli* DSM 1103 (ATCC 25922) indicated in the CLSI and EUCAST standards (33, 57).

### Transmission electron microscopy—TEM

Suspensions of gram-positive bacteria were prepared by taking bacterial colonies out of overnight grown agar plates and resuspending them in MHB. The suspension was set to an OD_625_ extinction of 0.15–0.20 corresponding to a bacterial cell density of approximately 2 × 10^8^ CFU/mL. Then, 3 × 1 mL of suspension was transferred into a 2-mL reaction tube and mixed with either plectasin, CASPON-plectasin, or PBS buffer to obtain volume conditions. The concentration for plectasin was 4 x MIC, and CASPON-plectasin was of the same molar quantity as plectasin. After incubation, the bacterial suspensions were washed with PBS three times, using mild centrifugation conditions for each wash cycle (3,000 *g*, 15 minutes) to avoid cell damage. Prior to sample preparation, mesh copper grids were pretreated with pioloform film and a thin carbon layer. Additionally, the grids were plasma-cleaned for 10 seconds. All samples were prepared by a negative staining procedure. Sample droplets were placed on copper grids for 10 minutes. Afterward, the grids were placed on a 2% glutaraldehyde in 0.1 M phosphate buffer, washed three times with HQ water, and stained with 1% uranyl acetate for 30 seconds. Grids were then analyzed in the TEM FEI Tecnai G^2^ 200 kV (Thermo Fisher Scientific, Waltham, MA) under high vacuum with 160 kV. For quantification, each condition (untreated, plectasin-treated, and CASPON-plectasin-treated) and each bacterial strain (the four mentioned above) were analyzed using two independent grid squares per biological duplicate, thus yielding a total of four grid squares per condition. Grid squares comprising ≥11 cells were selected for analysis, and the cells were counted and categorized as “no visible damage” or “visible damage”. Percentages and standard deviations were subsequently calculated for each condition. An example of counting the cells can be found in the supplementary material (Fig. S2).

### Atomic Force Microscopy—AFM

All AFM measurements were performed on a Vecoo-Bruker AFM (Diamond, Chilton, UK), including imaging and force distance measurement. Before and after measurements, the triangular cantilever was cleaned with EtOH and acetone. Sensitivity and the spring constant were calibrated by the thermal tune method by making use of the equipartition theorem, preceded by continued ramping. After calibration, the spring constant was set to 0.4017 N/m for performing measurements.

#### AFM sample preparation

Bacterial suspensions were prepared by taking bacterial colonies out of overnight grown agar plates and resuspending them in MHB. The suspension was set to an OD_625_ extinction of 0.15–0.20 corresponding to a bacterial cell density of approximately 2 × 10^8^ CFU/mL. Then, 3 × 1 mL of suspension was transferred into a 2-mL reaction tube and mixed with either plectasin, CASPON-plectasin, or PBS buffer to obtain volume conditions. The concentration for plectasin was 4 x MIC, and CASPON-plectasin was of the same molar quantity as plectasin. After incubation, the bacterial suspensions were washed with PBS three times, using mild centrifugation conditions for each wash cycle (3,000 *g*, 15 minutes) to avoid cell damage. The washed bacterial suspension was transferred to borosilicate glass slides (24 mm diameter, 0.1 mm thickness, Menzel Gläser, Braunschweig, Germany) and left to adhere to the surface (electrostatics) for 1 hour. Afterward, the slides were washed with MilliQ water and transferred to AFM.

#### AFM imaging

Bacterial images were visualized by square areas of 2 × 2 µm^2^ up to 25 × 25 µm^2^ with a resolution of 512 × 512 pixels. At least 15 images were taken per sample with different square dimensions for proper visualization. Surface roughness and shapes of the bacterial cell were estimated using Gwyddion software after applying a mean filter to the raw images. A total of 20 measurements were taken on cells for each parameter, providing enough values for statistical evaluation. For additional information and an illustration of the determination principle, please refer to the supplementary material (Fig. S3), which includes an example on *S. aureus*. By using the following equations, the surface roughness of a selected area of this flattened area was calculated from the standard deviation in the height image. The mean roughness (Ra) and the root-mean-square roughness (Rq or RMS) were used to characterize the surface of different samples:


(1) $$Ra=\frac{1}{N}\sum_{i-1}^{N}\left|Z_{i}\right|$$

(2) $$Rq=\sqrt{\sum_{i=1}^{n}\frac{{\left(Z_{i}-Z_{m}\right)}^{2}}{N-1}}$$

where N is the total number of data points, Z_i_ is the height of the i-th point, and Z_m_ is the mean height. A schematic drawing of the measured cell thickness and cell roughness parameters is visualized in the supplementary material (Fig. S4).

#### Force spectroscopy

After the position of the bacteria was determined, force spectroscopy was performed. Measurement of the central region of bacteria was performed with an approach speed of 10 µm/s and a maximum force of 2 nN. A curve length of 1 µm and a sampling rate of 2 kHz were used. At least 20 bacteria per sample were measured. Analysis of the force distance curves was performed using the software NanoScope Analysis 1.5. For calculating the stiffness of each bacterial cell, the following question was utilized:


(3) $$\frac{1}{k_{eff}}=\frac{1}{k_{sample}}+\frac{1}{k_{spring}}$$

where k_eff_ is the effective stiffness (received from the slope of the measured curve), k_sample_ is the stiffness of the sample (bacterial cell), and k_spring_ is the spring constant (0.4017 N/m). All constants have the unit N/m. Rearrangement of the formula leads to the following relation:


(4) $$k_{sample}=\frac{k_{spring}*k_{eff}}{k_{spring}-k_{eff}}$$

#### Statistics

Measurements were pooled, and properties were calculated as described above. For each parameter, 20 measurements were used to obtain appropriate statistical values. Analysis of variance (ANOVA) was performed, followed by Student’s *t*-test, which is a statistical procedure that compares the averages of two independent groups (plectasin/CASPON-plectasin treated vs untreated) to determine significance in the surface roughness and stiffness of bacteria. Significances are indicated in figures and reported as “ns” for *P* > 0.05, “*” for *P* ≤ 0.05, “**” for *P* ≤ 0.01, and “***” for *P* ≤ 0.001.

### Analytical methods

#### SDS-PAGE

Thirteen microliters of each sample was mixed with 5 µL LDS sample buffer (×4) (Invitrogen, Waltham, MA) and 2 µL reducing agent (×10). After heating those mixtures at 70°C for 10 minutes, 15 µL was loaded on an SDS-polyacrylamide NuPage 4%–12% Bis-Tris Gel, 1.0 mm (Invitrogen, Waltham, MA, USA). For molecular weight determination, SeeBlue Plus2 Pre-Stained Protein Standard (Thermo Fisher Scientific, Waltham, MA, USA) was used. Electrophoretic separations were performed for 45 minutes at 200 V (400 mA) in MES-SDS running buffer. After that, gels were incubated in fixing solution (50% ethanol and 10% acetic acid) for 30 minutes and then stained for 30 minutes in Coomassie staining solution (0.11% m/V Coomassie R250, 0.02% m/V Bismarck Brown R, 40% ethanol, and 10% acetic acid). After incubation in de-staining solution (25% ethanol and 8% acetic acid) for 2 hours, the gels were incubated in RO-H_2_O overnight prior to scanning. For all incubation steps, the gels were gently agitated by using an orbital shaker.

#### Peptide quantification

The same RP-HPLC method described by Müller *et al*. (24) was employed for CASPON-plectasin/plectasin quantification. For CASPON-plectasin and plectasin, the concentrations 1, 0.5, 0.25, 0.125, and 0.0625 g/L were utilized to construct the calibration curve.

#### Mass spectrometry

All mass spectrometry measurements were performed according to Müller *et al*. (24).

## RESULTS

### Purification of plectasin

**Fig 1:**
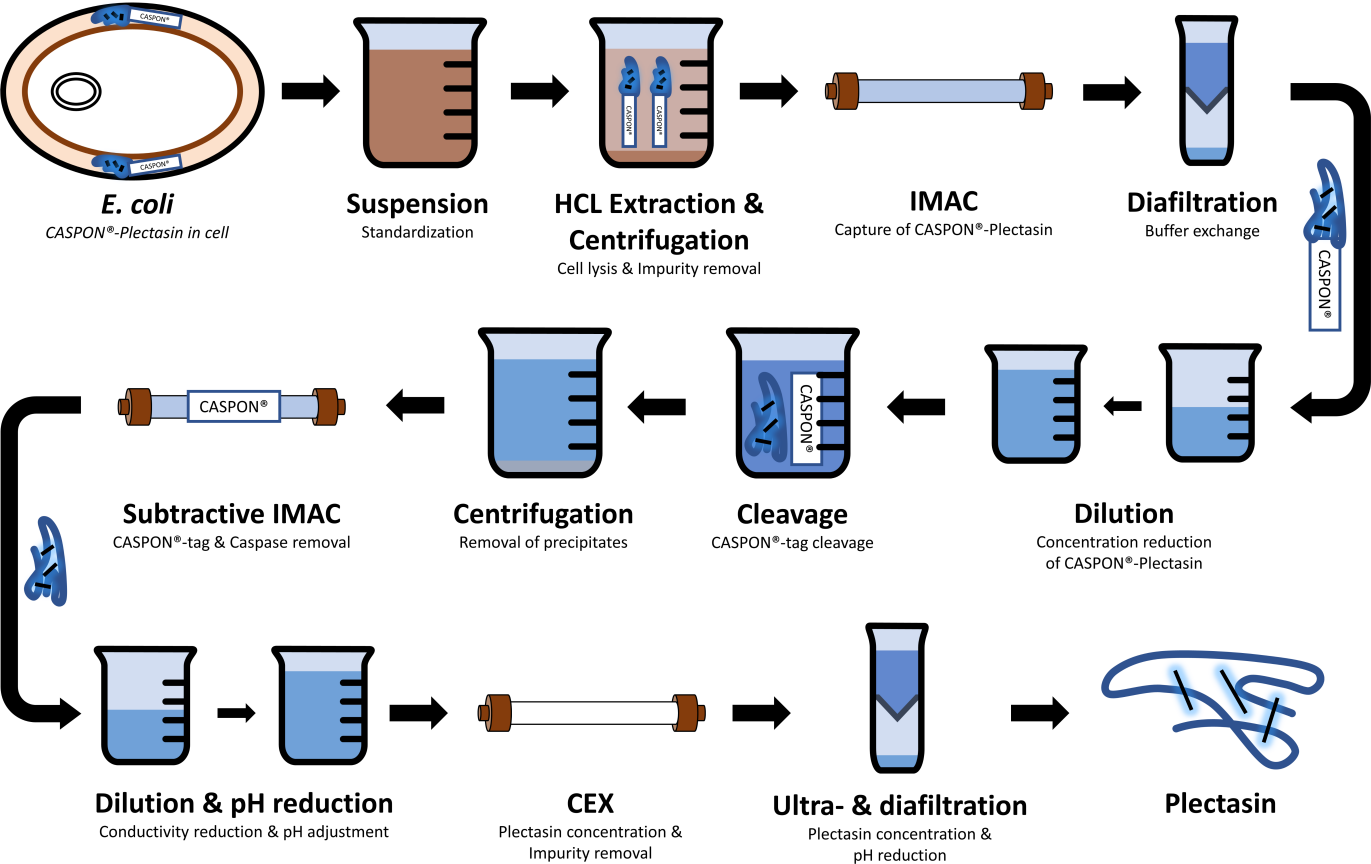
Schematic overview of the plectasin purification process, using the CASPON technology. After fermentation, the CASPON-tagged plectasin-producing cells are harvested and stored at −20°C. The process is initiated by resuspending the cells in buffer. Subsequent to cell extraction and centrifugation, CASPON-plectasin is captured via Immobilized Metal Affinity Chromatography (IMAC). The eluate of the captured product undergoes diafiltration and dilution to achieve the appropriate buffer conditions and concentration for CASPON enzyme-mediated tag cleavage. Subsequent centrifugation removes any impurity precipitate formed during the cleavage. The cleaved CASPON-tag and other negatively charged impurities are eliminated through a second IMAC step. The peptide-containing flow-through is then diluted, and the pH is adjusted for cation exchange chromatography (CEX). For specific experiments, the peptide solution is further adjusted in terms of buffer conditions and peptide concentration.

For purification, the CASPON platform process developed for the peptide SST was tailored to plectasin with minor modifications. Figure 1 illustrates the purification process, starting with the resuspension of the bacterial cells to the final ultra- and diafiltration step for the required formulation. An additional dilution step was incorporated into the protocol to prevent the plectasin concentration from exceeding 1.6 g/L after cleavage. As detailed in the supplementary material (Fig. S5), preliminary experiments demonstrated that under any selected cleavage condition, the highest achievable concentration is 1.5 g/L. The addition of 1 mM DTT as a supporting agent during the enzymatic CASPON-tag removal step enabled the increase in the plectasin concentration to 1.6 g/L. However, an increase in DTT resulted in a significant reduction in the yield, as illustrated in the supplementary material (Fig. S6). After dilution and an application of subtractive IMAC, the pH was reduced to enhance the positive charge of plectasin, thereby facilitating cation exchange (CEX) chromatography. Finally, the concentration and buffer systems were adjusted for activity assays and electron microscopy experiments, which included ultra- and diafiltration.

Fundamental monitoring including qualitative (SDS-PAGE) and quantitative (RP-HPLC) analyses of plectasin was conducted to assess the efficacy of the modified CASPON process. Chromatograms of all three chromatographic steps applied are provided in the supplementary material (Fig. S7).

**Fig 2:**
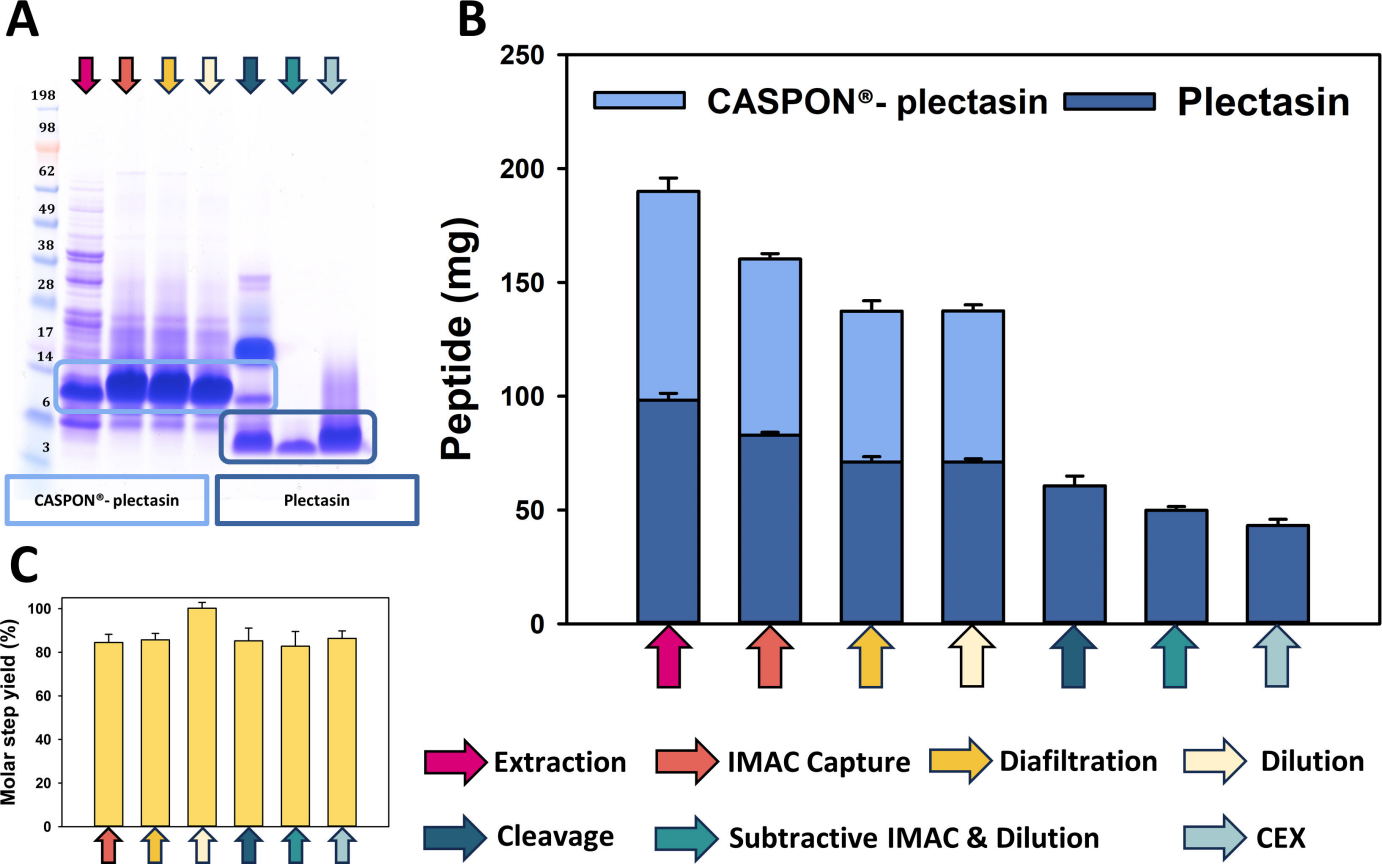
CASPON-plectasin purification monitoring of the most important process stages. Each purification step is indicated by arrows of a distinct color. (A) SDS-PAGE visualization of every purification step with undiluted samples. The peptides CASPON-plectasin and plectasin are highlighted by light and dark blue, respectively. (B) depicts the absolute CASPON-plectasin/plectasin content at the conclusion of each purification step. (C) represents the molar yields of the downstream steps. All values are given in mean ± standard deviation of *n* = 3 biological replicates.

The SDS-PAGE (Fig. 2A) confirmed the anticipated molecular weights for CASPON-plectasin and plectasin. Prior to dilution, the predominant band observed was in a range between 6 and 14 kDa, which aligns with the theoretical molecular weight of CASPON-plectasin (8.5 kDa). Following cleavage and subsequent purification, the main band representing plectasin remained between 3 and 6 kDa, which corresponds to the theoretical mass of plectasin, which is 4.4 kDa. Regarding HCPs, it can be observed that only the lane of the extract (pink arrow) and of the cleavage (dark green arrow) showed impurities, whereas the remainders appeared to be of a high degree of purity. Since some of the SDS-PAGE lanes are overloaded due to undiluted samples, an additional 1:10 dilution SDS-PAGE analysis was performed. This analysis, provided in the supplementary material (Fig. S8), clearly shows that only highly pure plectasin remains after this step, with no detectable impurities. The densities of the SDS-PAGE bands correlate well with the quantitative concentration analysis of RP-HPLC, for which the chromatograms are presented in the supplementary material (Fig. S9). However, it is important to note that the volumes differ after each purification step. The total peptide content was calculated using the peptide concentration and the captured volume after each purification step, with the resulting data presented in Fig. 2B. Furthermore, impurity estimation was conducted for each purification step using RP-HPLC chromatograms (Fig. S9), with peak analysis employed to monitor the purity throughout the process and to confirm high purity at the end. This is outlined in the supplementary material (Table S2). Mass spectrometry data of purified CASPON-plectasin, plectasin, and reduced plectasin are also shown in the supplementary material (Fig. S10). The concentrations, total quantities, and molar step yields are presented in Table 1 in numerical form. The molar step yields are, except for the dilution step, all approximately 85%.

**TABLE 1:** The data monitoring of the purification pathway included the measurement of the peptide concentration (CASPON-plectasin/plectasin), absolute peptide content, and molecular yield at each purification step.^*c*^

Purification step	Peptide (mg/mL)	Peptide (mg)	Mol yield (%)
Extraction	1.0 ± 0.0^*a*^	190.0 ± 5.9^*a*^	^*d*^-
IMAC capture	5.3 ± 0.1^*a*^	160.3 ± 2.4^*a*^	85 ± 4
Diafiltration	4.6 ± 0.2^*a*^	137.4 ± 4.5^*a*^	86 ± 3
Dilution	3.2 ± 0.1^*a*^	137.5 ± 2.6^*a*^	100 ± 5
Cleavage	1.4 ± 0.1^*b*^	60.6 ± 4.3^*b*^	85 ± 6
Subtractive IMAC & Dilution	0.7 ± 0.0^*b*^	49.9 ± 1.5^*b*^	83 ± 4
CEX	2.0 ± 0.1^*b*^	43.1 ± 2.7^*b*^	86 ± 3

^*a*^ CASPON-plectasin.

^*b*^ Plectasin.

^*c*^ All values were expressed as the mean ± standard deviation of *n* = 3 biological replicates.

^*d*^ "-" means that no purification yield has been determined.

The overall yield of the process, calculated by multiplying the step yields, is 45%.

### Evaluation of antimicrobial activity

It is necessary to ascertain whether plectasin has the same activity when fused to the CASPON-tag. In this instance, the purification process was terminated at the diafiltration stage, resulting in a more valuable overall yield of 73%. The purity of the CASPON-plectasin after the diafiltration step was found to be relatively high at 80.4% (Table S2), which is considered to be sufficient for certain applications. To verify this assertion of active CASPON-plectasin, we established an activity assay using microtiter plates that enables the determination of MICs through photometric measurement of bacterial growth. We investigated not only the activity of plectasin against several gram-positive and gram-negative bacteria but also of CASPON-plectasin to evaluate the impact of the negatively charged CASPON-tag.

**Fig 3:**
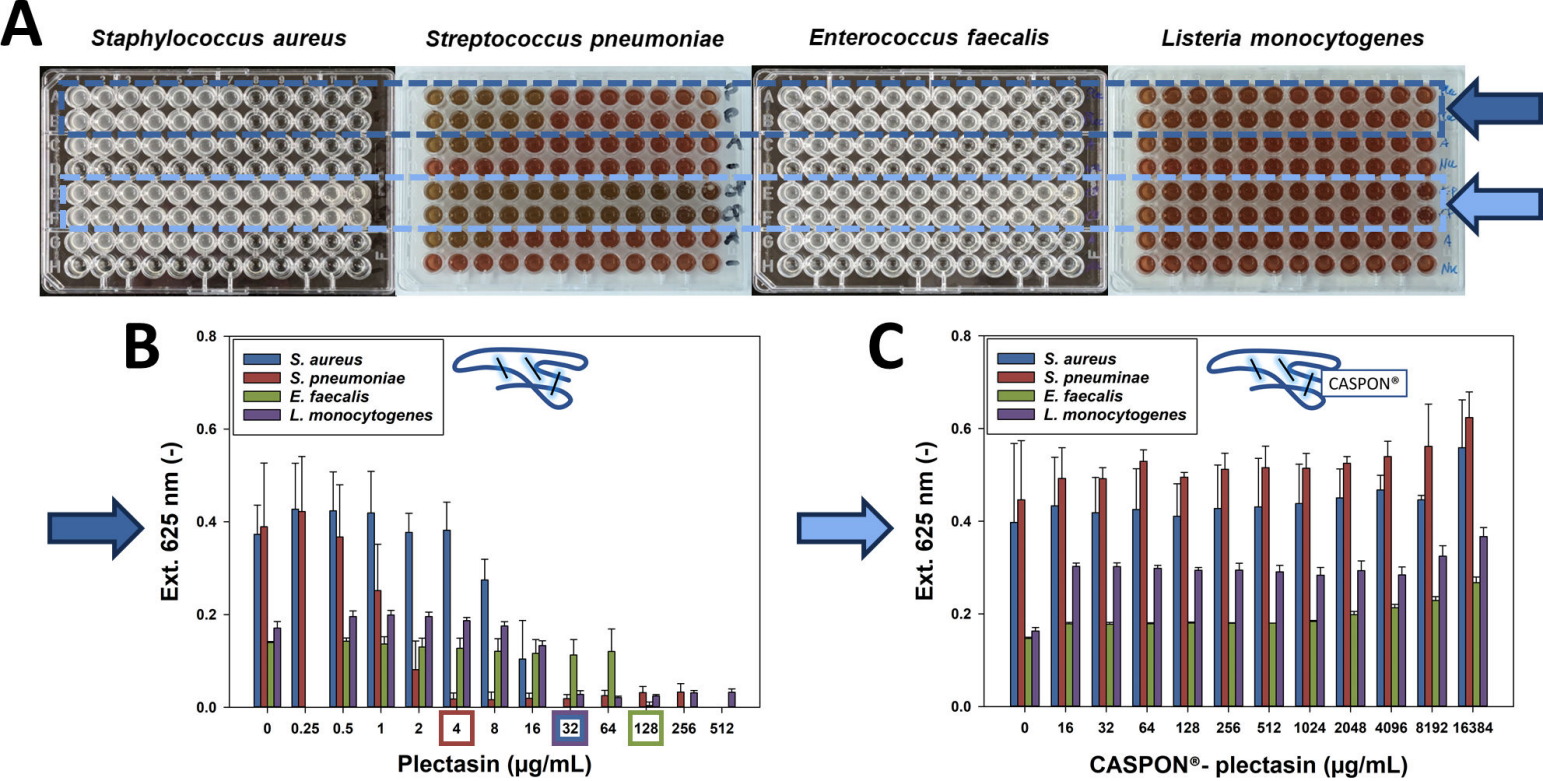
Antimicrobial activity assay for gram-positive bacteria. Microdilution plates of the bacteria *Staphylococcus aureus*, *Streptococcus pneumoniae*, *Enterococcus faecalis,* and *Listeria monocytogenes* are shown in the first row. The dark and light blue arrows indicate the plectasin and CASPON-plectasin concentration gradients, respectively. The corresponding UV–Vis absorbance plots are shown below. The x-axis describes the concentration of the peptide, while the y-axis represents the measured extinction at 625 nm. The data regarding extinction values are presented as a mean plus standard deviation error bars, obtained from three independent microdilution tests. The colored rectangles highlight the MIC concentrations obtained.

To facilitate the comparison with other MIC data for the purpose of proper validation, we compared the activity of plectasin, CASPON-plectasin, and ampicillin on one microdilution plate. The influence of cell growth was investigated using four different gram-positive bacteria (*Staphylococcus aureus*, *Streptococcus pneumoniae*, *Enterococcus faecalis*, and *Listeria monocytogenes*) and two gram-negative bacteria (*Pseudomonas aeruginosa* and *Escherichia coli*). The latter was tested in three different subclasses. In order to avoid the introduction of statistically significant errors, each bacterium was tested on three separate occasions. Figure 3 depicts the activity assay plates of gram-positive bacteria and their respective extinction values at a wavelength of 625 nm.

**Fig 4:**
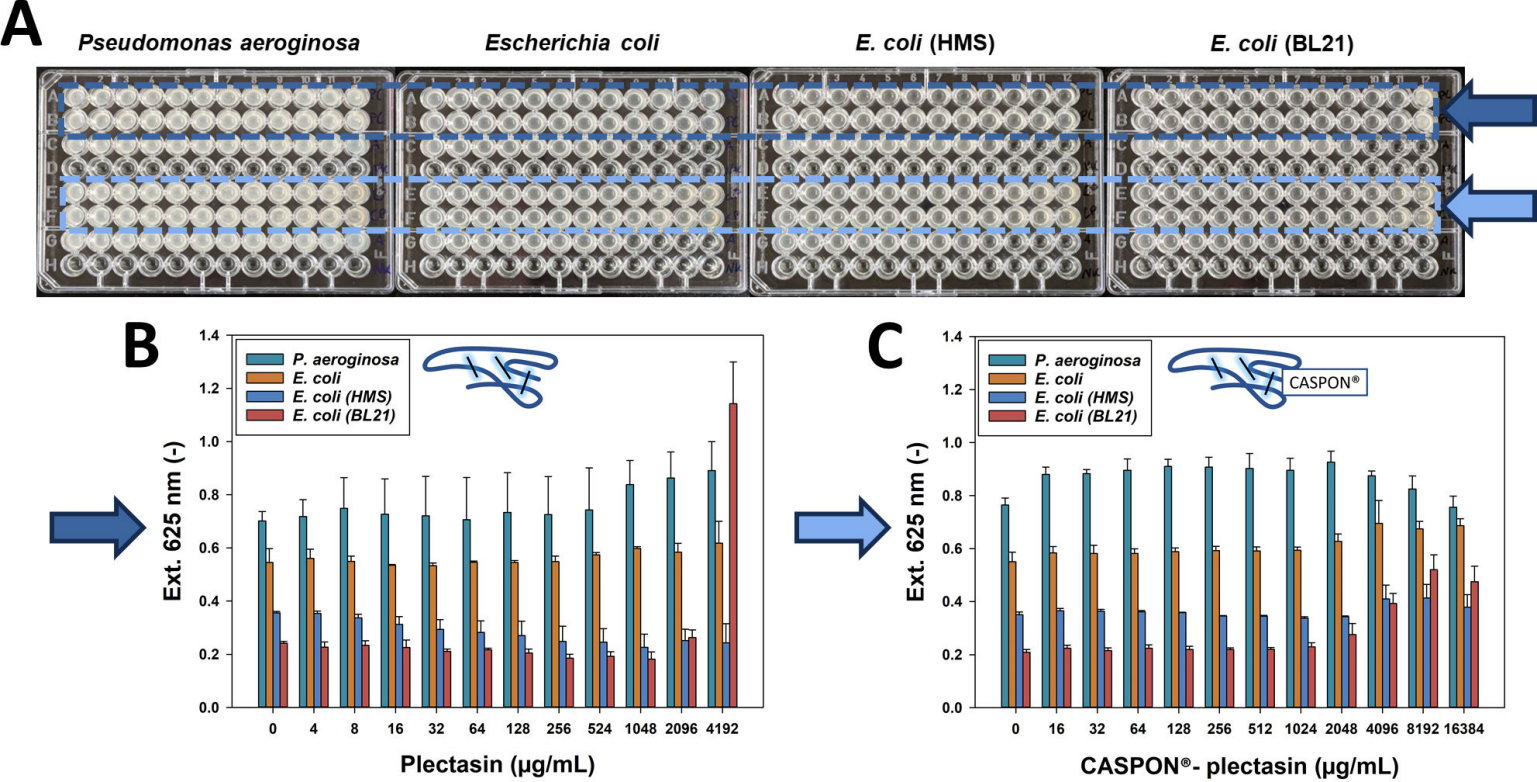
Activity test used for gram-negative bacteria. Microdilution plates of the bacteria *P. aeruginosa, Escherichia coli, E. coli HMS, and E. coli BL21* are shown in the first row. The dark and light blue arrows indicate the plectasin and CASPON-plectasin concentration gradients, respectively. The corresponding UV–Vis absorbance plots are shown below. The x-axis describes the concentration of the peptide, while the y-axis represents the measured extinction at 625 nm. The data regarding extinction values are presented as a mean plus standard deviation error bars, obtained from three independent microdilution tests.

Note that the absolute extinction differs between each bacterial strain. In particular, *E. faecalis* and *L. monocytogenes* exhibited a 50% reduction in extinction compared to *S. aureus* and *S. pneumoniae*. Nevertheless, in all four instances, a plateau was reached at the non-inhibiting concentrations. As illustrated in the left diagram of Fig. 3, a distinct gradient of extinction reduction was observed with *S. aureus* and *S. pneumoniae*, at plectasin concentrations of 8 and 0.5 µg/mL, respectively. The MIC for *S. aureus* was 32 µg/mL, while no growth was observed for *S. pneumoniae* at concentrations of 4 µg/mL or above. The growth of *E. faecalis* and *L. monocytogenes* was abruptly inhibited at concentrations of 128 and 32 µg/mL, respectively, without exhibiting a gradual reduction in growth. In the case of CASPON-plectasin (Fig. 3C), no growth inhibition was observed, even at a high concentration of 16,384 µg/mL. Slightly higher extinction values were measured at high concentrations, which is due to enhanced bacterial growth as a result of increased availability of nitrogen. Figure 4 represents the activity assay plates of gram-negative bacteria (*P. aeruginosa*, *Escherichia coli*, *E. coli HMS*, and *E. coli BL21*) and their respective extinction values at a wavelength of 625 nm.

Because gram-negative bacteria expose a reduced peptidoglycan layer in their membranes, they supposedly exhibit lower sensitivity to plectasin. Therefore, higher concentrations of plectasin were employed to ascertain the MIC. However, the solubility of plectasin is limited at neutral pH, which restricts the maximum achievable concentration to 4,192 µg/mL. In all four cases, the gram-negative bacteria demonstrated consistent growth patterns, irrespective of the plectasin concentration. It is noteworthy that *E. coli* BL21 exhibited an extinction value that was four times higher at 4,192 µg/mL. The supplementary material (Fig. S11) contains microscopic images of this phenomenon, which demonstrate an increased number and altered morphology of *E. coli* BL21. In the case of CASPON-plectasin, growth was observed at all concentrations, as illustrated in the right diagram of Fig. 4C. Furthermore, higher concentrations resulted in marginally increased extinction values and, consequently, increased growth, except for *P. aeruginosa*, which exhibited a slight decline. However, neither plectasin nor CASPON-plectasin demonstrated any inhibitory effects on the growth of the gram-negative bacteria. Consequently, no MICs could be determined. Table 2 provides a summary of the MICs obtained. All gram-positive bacteria exhibited a reduction in growth when treated with plectasin. In contrast, gram-negative bacteria showed no growth decline when treated with plectasin. Overall, treatment with CASPON-plectasin did not result in any observable inhibition of bacterial growth.

**TABLE 2:** Minimum inhibitory concentration of monitored bacteria^*a*^

	MIC (µg/mL)			
Gram-positive bacteria	*S. aureus*	*S. pneumoniae*	*E. faecalis*	*L. monocytogenes*
Plectasin	32	4	128	32
CASPON-plectasin	ND	ND	ND	ND
Gram-negative bacteria	*P. aeruginosa*	*E. coli*	*E. coli HMS*	*E. coli BL21*
Plectasin	ND	ND	ND	ND
CASPON-plectasin	ND	ND	ND	ND

^*a*^ ND, not determined.

### TEM measurements

**Fig 5:**
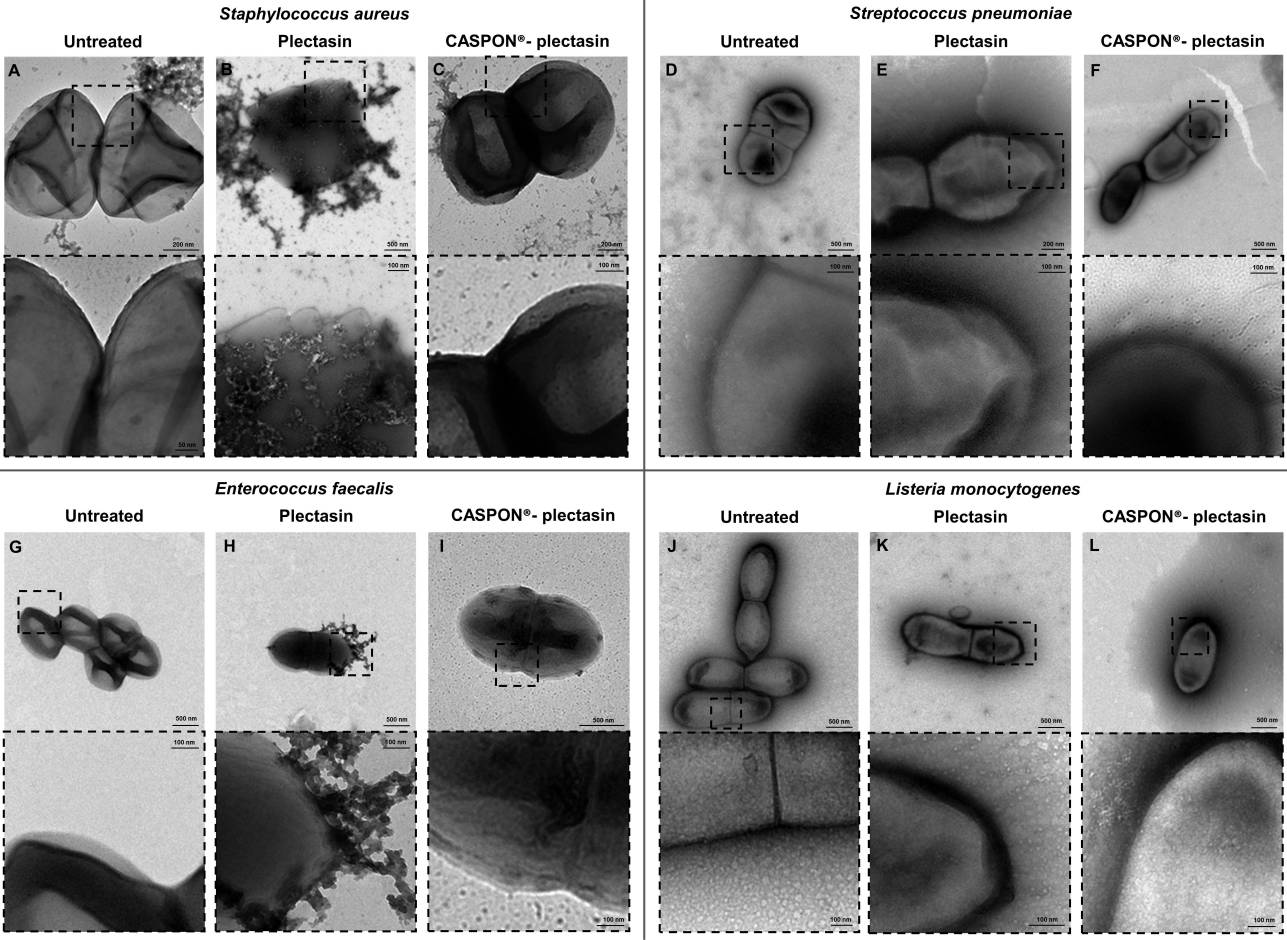
The transmission electron microscopy measurements of the gram-positive bacteria *Staphylococcus aureus*, *Streptococcus pneumoniae*, *Enterococcus faecalis*, and *Listeria monocytogenes*. The images of the untreated, plectasin-, and CASPON-plectasin-treated bacteria are shown in the first, second, and third columns, respectively. The dashed rectangles in the first row of each bacterium indicate the magnified region in the second row.

The morphological transformation of the four gram-positive bacteria that were impacted by plectasin was investigated via TEM. The microscopic images of the untreated, CASPON-plectasin-, and plectasin-treated *S. aureus*, *S. pneumoniae*, *E. faecalis,* and *L. monocytogenes* cells are presented in Fig. 5.

**Fig 6:**
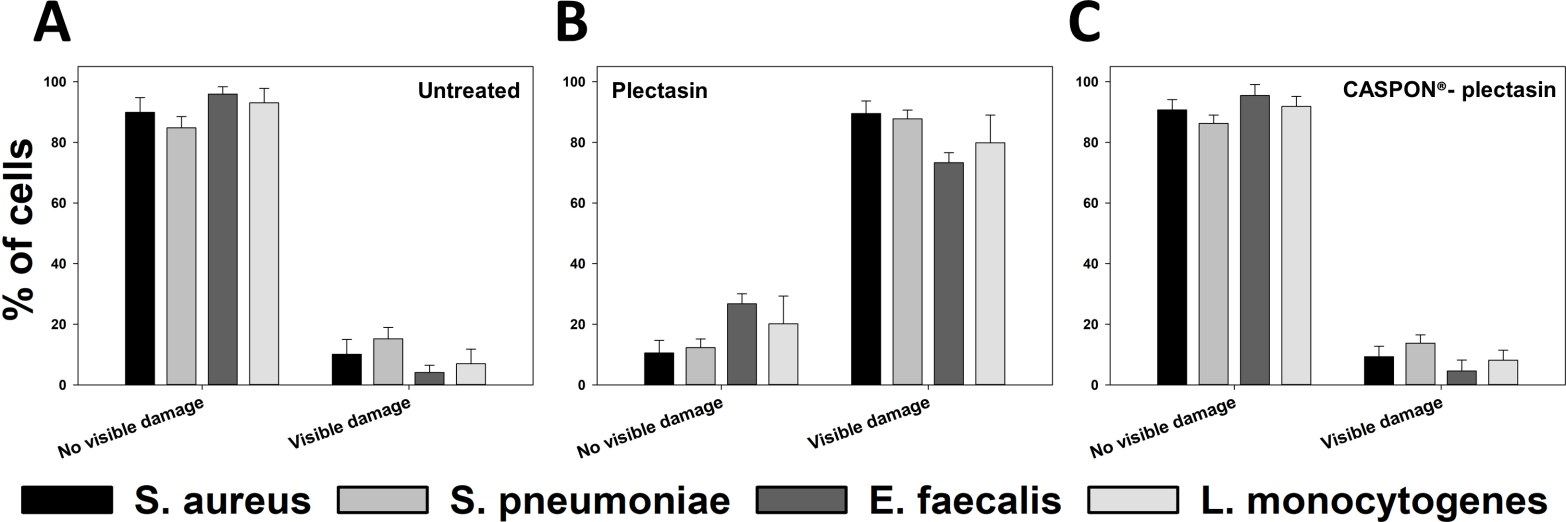
Quantitative analysis of transmission electron microscopy measurements of the gram-positive bacteria *Staphylococcus aureus*, *Streptococcus pneumoniae*, *Enterococcus faecalis,* and *Listeria monocytogenes*. Figures (A), (B), and (C) illustrate the percentage of undamaged and damaged cells after treatment with no agent, plectasin, and CASPON-plectasin, respectively. Cells were quantified manually from electron micrographs ranging from 5,000x to 11,500x magnification according to their phenotype. The analysis was performed on four grid squares from two biological replicates (2 × 2), yielding a minimum of 67 analyzed cells per condition. The data represent the mean ± standard deviation of the percentages calculated from the four analyzed grid squares per condition.

The four strains that were not subjected to peptide treatment during the growth phase displayed intact shapes and no indications of damage to the membrane structure (Fig. 5A, D, G and J). Magnifications revealed the presence of smooth surfaces and morphologically stable structures. Quantitative analysis, as shown in the accompanying diagram (Fig. 6A), indicated that the percentage of cells with no visible damage was high across all strains: 89.90% ± 4.88% for *S. aureus*, 84.80% ± 3.71% for *S. pneumoniae*, 95.93% ± 2.41% for *E. faecalis,* and 93.03% ± 4.81% for *L. monocytogenes*.

Treatment with plectasin during the growth phase resulted in visible damage to cells, although the extent of this damage varied between the different bacteria. In the case of *S. aureus* treated with plectasin (Fig. 5B), clear spikes were formed on the surface. Magnification revealed the presence of crystal structures on the surface, indicating incomplete peptidoglycan layers. Quantitatively, 89.50% ± 4.15% of *S. aureus* cells exhibited visible damage (Fig. 6B). Additionally, *S. pneumoniae* exhibited incomplete cell structures (Fig. 5E). Upon closer examination, the cells in Fig. 5E displayed a distinctive pattern of crater-like depressions on their surface, rather than the spikes, with 87.75% ± 2.90% of cells showing visible damage. *E. faecalis* exhibited minimal structural alterations, with 73.30% ± 3.31% of cells showing damage. Figure 5H illustrates the presence of discrete regions where cytoplasm extrudes from the cell. *L. monocytogenes* showed comparable morphological integrity to that of *S. pneumoniae*, although to a lesser degree (Fig. 5K), with 79.87% ± 9.13% of cells exhibiting visible damage.

In the case of cells treated with CASPON-plectasin, the morphological appearance was similar to that of untreated bacteria (Fig. 5C, F, I and L). However, the cells were slightly darker in overall appearance. The most notable change was seen in *E. faecalis*, where dark spots were visible on the bacterial surface (Fig. 5I), which may indicate the occurrence of cell repair mechanisms. Despite this observation, the magnification still showed a very smooth membrane surface. The quantitative analysis revealed that the percentage of cells with no visible damage was 90.71% ± 3.40% for *S. aureus*, 86.28% ± 2.75% for *S. pneumoniae*, 95.44% ± 3.63% for *E. faecalis,* and 91.86% ± 3.28% for *L. monocytogenes* (Fig. 6C).

Figure 6 confirms the visual observations made in the TEM images of the extent of cellular damage triggered by plectasin and CASPON-plectasin treatments. Detailed data on the number of cells examined per grid square are provided in the supplementary material (Table S3).

### AFM measurements

**Fig 7:**
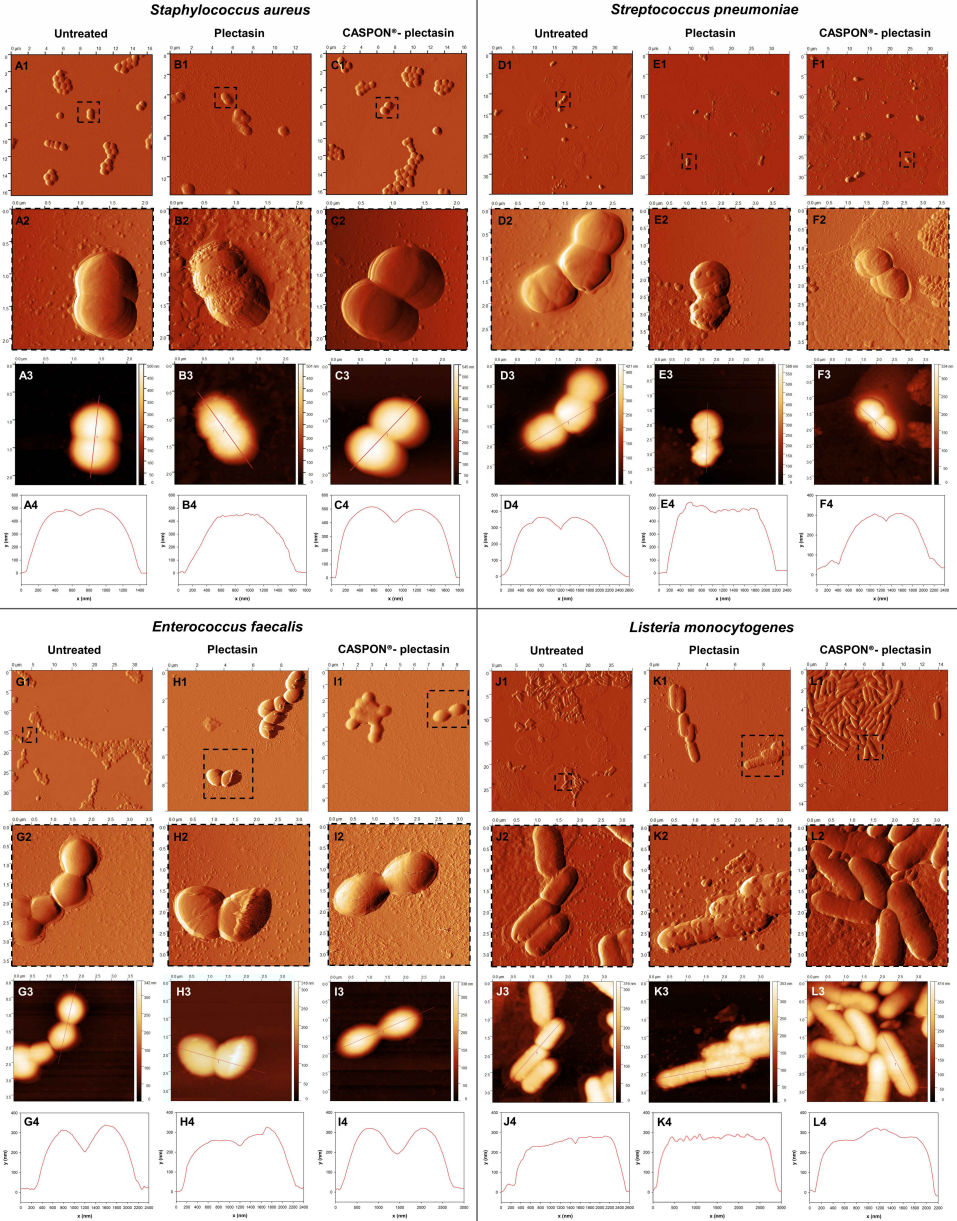
Atomic force microscopy measurements of the gram-positive bacteria *S. aureus* (A–C), *S. pneumoniae* (D–F), *E. faecalis* (G–I), and *L. monocytogenes* (J–L). The images of the untreated, plectasin-, and CASPON-plectasin-treated bacteria are presented in column 1-3 for each bacteria. Row 1 depicts overview images of the bacteria from the deflection error image, and row 2 displays magnifications of the regions indicated in row 1. Row 3 shows height images, and row 4 the topographic profile obtained from the height images.

AFM microscopy was performed to investigate the microbial surfaces at high resolution of the four selected gram-positive bacteria. AFM allows for quantifiable statements on the cellular morphology at the single-cell level. By applying a force, the cantilever can determine the height and measure the stiffness of an object. Figure 7 provides a summary of AFM images of selected gram-positive bacteria that were untreated, treated with plectasin, and treated with CASPON-plectasin. The untreated bacteria exhibited smooth cell shapes in all four strains. Further magnification (presented in dashed-line frame images) further emphasizes this observation. The height images (Fig. 7A3, D3, G3 and J3) and their corresponding topographical profiles (Fig. 7A4, D4, G4 and J4) serve to corroborate the vitality of the cells in an even more compelling manner.

In contrast, treatment with plectasin resulted in the deformation of the cell membrane, thereby compromising its structural integrity. In all four cases of bacterial strains, the decay of the peptidoglycan layer was visible, although the damage appeared to be distinct in each instance. In contrast to the formation of small spikes observed in *S. aureus* (Fig. 7B2), *S. pneumoniae* and *L. monocytogenes* displayed protuberances on the bacterial surface (Fig. 7E2 and K2). The *E. faecalis* membrane appeared to be relatively unperturbed, exhibiting only slight deformations (Fig. 7H3). These observations are also reflected by the topographic profiles (Fig. 7B4, E4, H4 and K4). It is notable to mention that *E. faecalis* is the sole bacterium in which biofilms were identified. The data presented in Fig. 7G1 and L1 clearly demonstrate the formation of biofilms between cells in the bacterial piles. Treatment with plectasin resulted in the absence of biofilm formation, which suggests the existence of a stress response.

Gram-positive bacteria treated with CASPON-plectasin exhibited characteristics similar to those of the untreated bacteria. The unenlarged images (Fig. 7C1, F1, I1 and L1) show a comparable number of cells, and the topographical profiles (Fig. 7C4, F4, I4 and L4) did not differ from those of the untreated bacteria.

**Fig 8:**
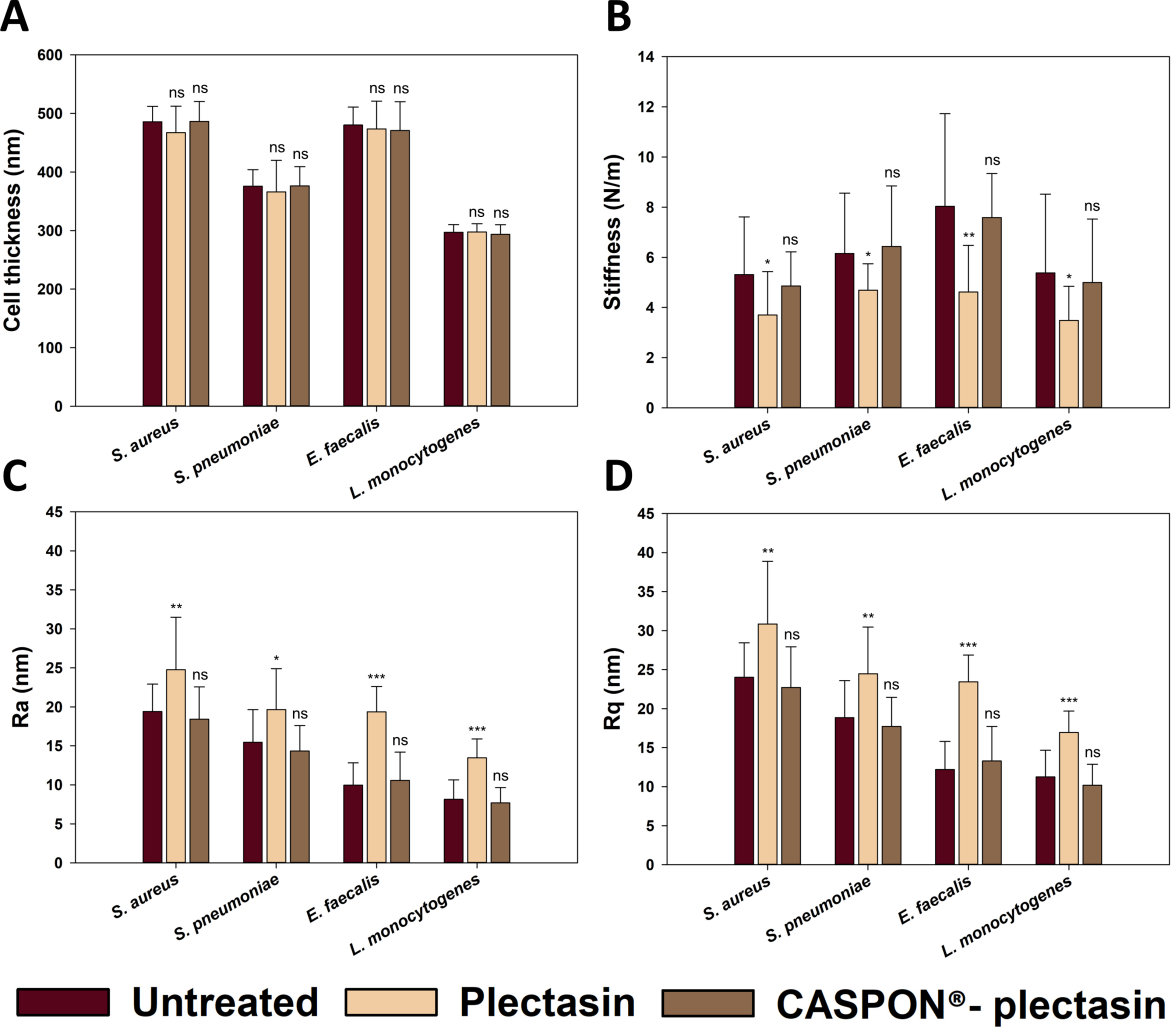
Cell thickness, stiffness, and roughness (Ra and Rq) of the gram-positive bacteria *Staphylococcus aureus*, *Streptococcus pneumoniae*, *Enterococcus faecalis,* and *Listeria monocytogenes*. Figure (A) illustrates the cell thickness, Figure (B) depicts the stiffness of the cells, and Figures (C) and (D) illustrate the roughness parameters Ra and Rq, respectively. The untreated, plectasin-treated, and CASPON-plectasin-treated bacterial cells are represented by dark red, beige, and brown columns, respectively. The data represent the mean ± standard deviation of 20 measurements. Significances are indicated in figures and reported as not significant “ns” for *P* > 0.05, “*” for *P* ≤ 0.05, “**” for *P* ≤ 0.01, and “***” for *P* ≤ 0.001.

In order to confirm whether plectasin induces morphological alterations in vital bacteria, it is essential to undertake a quantitative analysis. Therefore, the cell thickness, cell stiffness, and roughness (Ra and Rq) of the bacteria following treatment with plectasin and CASPON-plectasin were determined. To ascertain the statistical significance, the means of 20 cells were calculated for each data set based on the recorded height images. Figure 8A demonstrates that neither plectasin nor CASPON-plectasin treatment resulted in a reduction in the mean cell thickness of gram-positive bacteria. No statistically significant difference was observed following the application of Student’s *t*-test.

As illustrated in Fig. 8B, the untreated bacterial strains exhibited slight differences in stiffness. However, after plectasin treatment, the stiffnesses of all bacterial cells tested were approximately 4 N/m. These stiffnesses demonstrate a statistically significant decrease of approximately one-third in all bacterial strains. The application of CASPON-plectasin resulted in no notable alteration in the observed stiffness, with calculated means highly comparable to those of the untreated bacteria.

The degree of roughness was assessed using the arithmetic mean of the roughness profile (Ra) (Fig. 8C) and the root mean square deviation (Rq) (Fig. 8D). These two parameters yielded comparable results, with Rq exhibiting slightly higher values in all cases. However, the plectasin treatment caused a twofold increase in roughness among all gram-positive bacteria. The observed values were statistically significant, with those for *E. faecalis* and *L. monocytogenes* exhibiting particularly strong statistical significance. As with cell stiffness, CASPON-plectasin treatment did not result in any changes to the roughness parameters.

Table 3 provides a summary of the collected data, expressed numerically, for a more comprehensive understanding of the measured AFM parameters. The parameters strongly depend on the bacterial strain. However, the proportion of increase in stiffness and decrease in roughness appears to be consistent across all bacterial strains measured. Overall, the numerical quantities of standard deviations appear randomly distributed, suggesting statistically robust data.

**TABLE 3:** Numerical representation of gram-positive bacteria’s cell thickness, stiffness, and roughness (Ra and Rq)^*a*^

Bacteria	*S. aureus*	*S. pneumoniae*	*E. faecalis*	*L. monocytogenes*
Cell thickness (nm)				
Untreated	485.9 ± 26.5	375.8 ± 28.4	480.3 ± 30.6	297.0 ± 13.3
Plectasin	467.2 ± 45.1	366.0 ± 54.1	473.5 ± 47.5	297.8 ± 14.0
CASPON-plectasin	486.5 ± 33.9	376.4 ± 32.7	471.0 ± 49.2	293.6 ± 16.5
Stiffness (N/m)				
Untreated	5.3 ± 2.3	6.2 ± 2.4	8.0 ± 3.7	5.4 ± 3.1
Plectasin	3.7 ± 1.7	4.7 ± 1.1	4.6 ± 1.9	3.5 ± 1.4
CASPON-plectasin	4.9 ± 1.4	6.4 ± 2.4	7.6 ± 1.8	5.0 ± 2.5
Ra (nm)				
Untreated	19.4 ± 3.5	15.5 ± 4.2	10.0 ± 2.5	8.1 ± 2.5
Plectasin	24.76 ± 6.7	19.7 ± 5.3	19.4 ± 3.2	13.5 ± 2.4
CASPON-plectasin	18.4 ± 4.1	14.3 ± 3.3	10.6 ± 3.6	7.7 ± 2.0
Rq (nm)				
Untreated	24.0 ± 4.4	18.8 ± 4.8	12.2 ± 3.4	11.2 ± 3.4
Plectasin	30.9 ± 8.0	24.5 ± 6.0	23.5 ± 3.4	16.9 ± 2.8
CASPON-plectasin	22.7 ± 5.2	17.7 ± 3.7	13.3 ± 4.4	10.2 ± 2.7

^*a*^ The data represent mean ± standard deviation of 20 measurements.

## DISCUSSION

The production of antibiotic substances is very important to combat the threat of evolving multiresistant bacteria (58). The discovery of peptides capable of attacking infectious bacteria (35), coupled with economical and efficient manufacturing as shown in our recent studies (23, 24) on the peptide SST, represents crucial contributions to the development and characterization of AMPs.

Accordingly, the study herein focused on two main objectives. First, we wanted to verify that peptides produced using the CASPON platform process (23, 24) retain their structural integrity to effectively combat the pathogens. Second, the study investigated the impact of plectasin treatment on the morphology of gram-positive bacteria, specifically its effect on the synthesis and maintenance of the peptidoglycan layer.

The CASPON technology previously used for the production of SST (24) was tailored to plectasin. Only minor modifications in the purification process were necessary to achieve a high amount of plectasin. One of the modifications was to adjust the initial CASPON-plectasin concentration to 3.2 g/L in the CASPON enzyme-based tag removal step due to the lower solubility of plectasin (~1.6 g/L). The preliminary experiments presented in the supplementary material (Fig. S5 and S6) confirm published findings (12, 59), which indicate that the maximum solubility of plectasin is approximately 1.6 g/L. Another alteration was to reduce the pH to 6.0 prior to the CEX step, due to the lower pI of 7.77. A reduction in pH was required to enhance the binding capacity of the Capto S ImpAct column used. These modifications resulted in an enhanced outcome compared to the purification of SST. The cleavage process yielded 85% of the desired product, which is a typical yield for a downstream step and an improvement over the 50% yield obtained for SST. The required pH reduction resulted in a comparable yield for the CEX step. For each downstream step, step yields averaged approximately 85%, a value consistent with that found in other literature (60, 61). Summing up the step yields, the final overall yield for plectasin was 45%, representing a notable improvement over the 34% observed for SST. The overall yield of 45% is highly satisfactory, particularly in view of eight purification steps and the low level of impurities present at the end of the process. This illustrates that although the CASPON platform process needs adjustment to the peptide of interest, the downstream process can be easily tailored to each specific peptide. Ultimately, it was demonstrated that the CASPON technology facilitates the efficient production of plectasin in *E. coli* in comparison to other existing methods found in the literature, which yielded lower total yields with 12% (62) or 20% (63), and did not account for exact impurity levels.

To serve as an AMP, it is essential that the manufacturing process of the recombinant peptide retains its biological activity. Consequently, we devised a statistically robust methodology for determining the MIC of peptides. The results of the testing demonstrated that the MIC of the tested gram-positive bacteria (4–128 µg/mL) was comparable to that reported in the literature (12–14). As expected, the tested gram-negative bacteria exhibited no growth inhibition. It is noteworthy that at high concentrations of plectasin, both *P. aeruginosa* and *E. coli* demonstrated enhanced growth, likely due to plectasin serving as a protein source. The beneficial impact of increased protein levels is evidenced by multiple studies (64, 65).

To ascertain whether removal of the tag was necessary for the functionality of plectasin, the same susceptibility test was performed with the tagged plectasin, CASPON-plectasin. Omitting the enzymatic tag removal step would result in shortening of the entire DSP. The results demonstrated that neither the gram-negative nor the gram-positive bacteria exhibited growth inhibition, even at the highest concentration tested (16 mg/mL). It has been demonstrated that protein fusion tags can reduce and, in some cases, abolish the activity of a protein (66–68). Therefore, it can be postulated that the CASPON-tag may act to shield the active site of plectasin. The small size of plectasin may play a role in this process as the negatively charged CASPON prevents the active site from performing its function due to its proximity. Therefore, it is reasonable to assert that the CASPON-tag effectively inactivates peptides that could potentially compromise the viability of the *E. coli* production host. On the other hand, the results of the susceptibility tests suggest that the CASPON purification platform is a reliable approach to produce high quantities of biologically active peptides for subsequent investigation of newly discovered moieties.

The effect and efficiency of such antibiotic substances on bacteria would be a valuable area of study for the development of new drugs (69, 70). To this end, it is necessary to investigate the manner and extent to which such substances affect the pathogen. In the present example, a visual examination via TEM was conducted on four gram-positive bacteria following treatment with plectasin and CASPON-plectasin. Subsequently, a statistical evaluation was performed on the morphological alterations (e.g., cell thickness, stiffness, and roughness) of the bacteria via AFM, a methodology that had not previously been employed in this context with gram-positive bacteria. As anticipated, TEM revealed the presence of visible spikes and holes on the bacterial membrane surface following plectasin treatment. In particular, *S. aureus* cells exhibited clear spikes and hole-like structures on the membrane, while *S. pneumoniae* displayed crater-like depressions. In contrast, *E. faecalis* exhibited minimal structural alterations. *L. monocytogenes* demonstrated comparable morphological integrity to that of *S. pneumoniae*, though to a lesser degree. CASPON-plectasin did not cause significant membrane damage, which is consistent with the results of the susceptibility testing conducted previously. However, it was observed that *E. faecalis* exhibited black dots on the surface, indicating the presence of CASPON-plectasin accumulation or instances of cell repair mechanisms. This observation aligns with the findings of previous studies that demonstrated alterations in peptidoglycans after the exposure of bacterial cells to mild stress conditions (71, 72). It is noteworthy that *E. faecalis* and *L. monocytogenes*, which exhibit the highest stability in both untreated and CASPON-plectasin-treated conditions, also demonstrated relatively higher stability when subjected to plectasin treatment. Overall, the bacterial membrane stability is maintained during treatment with CASPON-plectasin stability similar to untreated bacteria, whereas all bacterial strains exhibited significantly reduced stability when treated with plectasin.

In addition to TEM, AFM was employed to quantify critical cellular parameters after exposure to plectasin or CASPON-plectasin, in comparison to untreated bacteria. It is notable that the cell thickness did not reduce in all instances, whether treated with plectasin or CASPON-plectasin (Fig. 8A). Conversely, plectasin treatment resulted in an increase in the surface roughness, as indicated by the Ra and Rq values (Fig. 8C and D). This is also evident in the images displayed in the topographic profiles of Fig. 7. Therefore, it can be concluded that the damage and decay of the peptidoglycan layer do not occur in a gradual, layer-by-layer manner. Instead, the formation of grooves in the membrane is the predominant phenomenon. This finding is consistent with that of a recently published study (22) which reported the formation of dense supramolecular structures on bacterial membranes by plectasin. These concentrated plectasin spots appear to cause the degradation of the peptidoglycan layer at specific locations. The observed reduction in stiffness (Fig. 8B) provides further evidence of this phenomenon. The resulting higher roughness and lower stiffness differed between treated and control bacteria, but the percentual increase and decrease were equivalent. The observation that all bacteria exhibited this alteration leads to the conclusion that plectasin does not only disrupt the synthesis of new peptidoglycans, as is claimed in the majority of studies (19, 20), but it also affects existing murein layers, at least it interferes with cell membrane repair mechanisms. This is supported by other works found in the literature (21, 22), which state that plectasin can disrupt the existing peptidoglycan layer. However, treatment with CASPON-plectasin did not disrupt the peptidoglycan layer, as demonstrated by the absence of alterations in roughness and stiffness.

In conclusion, the CASPON platform technology can be employed and adapted for the effective purification of peptides. Following the verification of the purification of the biochemically correct molecule SST in our previous study (24), this work demonstrates that the purified peptide plectasin was biologically active. Therefore, it can be concluded that the CASPON purification process preserves all three disulfide bridges in plectasin, which are necessary for its activity. The CASPON tag, used during purification, has been shown to fully inhibit the activity of plectasin. The highest concentration of CASPON-plectasin tested did not show any inhibitory effect on bacterial growth. This finding may be interesting with regard to other AMPs produced by the CASPON platform that could potentially harm the *E. coli* production host. After the successful production of plectasin, microscopic monitoring of its activity was conducted. The data obtained regarding cell thickness, stiffness, and roughness proved to be beneficial and should serve as a foundation for further studies on AMPs, which undoubtedly represent one of the most promising weapons against the growing threat of multiresistant bacteria.

Based on our study, we assume that the CASPON technology can be used in the future to produce any AMP suitable for medical applications as the CASPON-tag prevents antimicrobial activity in the producing cells, and only minor adjustments to the intrinsic properties of the peptides are required during production.

## Data Availability

The authors confirm that the data supporting the findings of this study are available for review within the paper and its supplementary information files. Should any raw data files be required in an alternative format, they are available from the corresponding author upon reasonable request.

## SUPPLEMENTAL MATERIAL

Supplemental tables and figures jb.00456-24-s0001.pdf Tables S1 to S3 and Figures S1 to S11.

ASM does not own the copyrights to Supplemental Material that may be linked to, or accessed through, an article. The authors have granted ASM a non-exclusive, world-wide license to publish the Supplemental Material files. Please contact the corresponding author directly for reuse.
