# Supplementary material for: Antimicrobial peptide plectasin recombinantly produced in Escherichia coli disintegrates cell walls of gram-positive bacteria, as proven by transmission electron and atomic force microscopy
Source: J Bacteriol. 2025 Apr 4;207(5):e00456-24. doi: 10.1128/jb.00456-24 (PMC12096834; doi:10.1128/jb.00456-24)
Supplement: Supplemental tables and figures — Tables S1 to S3 and Figures S1 to S11. [file jb.00456-24-s0001.pdf]

Antimicrobial peptide plectasin recombinantly produced in *Escherichia coli* disintegrates cell walls of gram-positive bacteria, as proven by transmission electron and atomic force microscopy

Authors: Matthias Müller[1], Sigrid Mayrhofer[2], Wisnu Arfian A. Sudjarwo[3], Martin Gibisch[1], Christopher Tauer[2], Eva Berger[1], Cécile Brocard[4], José L. Toca-Herrera[3], Gerald Striedner[1], Rainer Hahn[1] and Monika Cserjan-Puschmann[1]

Affiliation:
1 Christian Doppler Laboratory for production of next-level biopharmaceuticals in *E. coli*, Institute of Bioprocess Science and Engineering, BOKU University, Muthgasse 18, A-1190 Vienna, Austria
2 Institute of Molecular Biotechnology, BOKU University, Muthgasse 18, A-1190 Vienna, Austria
3 Institute of Biophysics, BOKU University Muthgasse 18, A-1190 Vienna, Austria
4 Boehringer Ingelheim RCV GmbH & Co KG, Dr.-Boehringer-Gasse 5-11, A-1120 Vienna, Austria

Keywords: recombinant peptides, *E. coli*, CASPON®-tag, purification, disulfide bridges

1 Supplementary

1.1 Amino acid sequences

Table S1 Amino acid sequences of peptides and signal sequence used in this study. Molecular weights were determined using the ExPASy online tool, assuming complete disulfide bond formation. OmpA: outer membrane protein A, SS: signal sequence, MW: molecular weight.

Peptide	Amino acid sequence	MW (Da)
$OmpA^{SS}$	MKKTAIAIAVALAGFATVAQA	2046.50
CASPON®-tag	LEDPERNKERKEAELQAQTAEQHHHHHHGSGVDVAD	4136.34
Plectasin	GFGCNGPWDEDDMQCHNHCKSIKGYKGGYCAKGGFVCKCY	4399.77

1.2 Scheme of activity assay

Figure S1 represents a scheme of the developed activity assay.

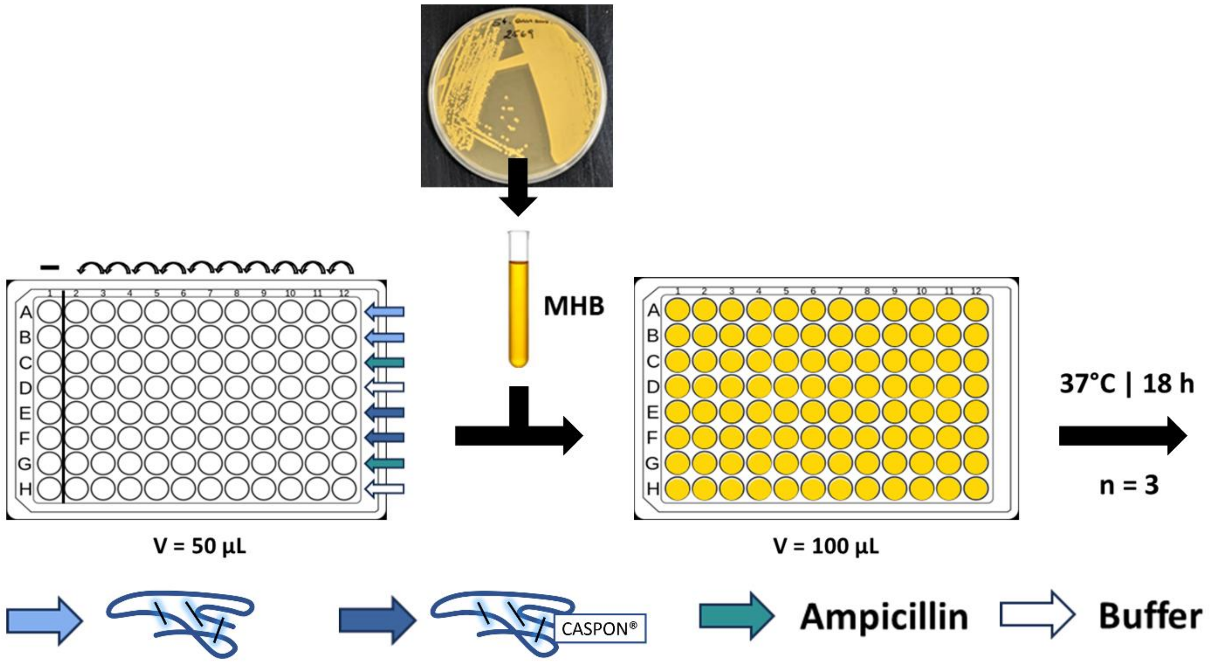

Figure S1 Schematic representation of the activity assay. Plectasin, CASPON®-plectasin, ampicillin and buffer are represented by light blue, dark blue, green and white arrows, respectively. MHB stands for Müller-Hinton-Bouillon, which was used to prepare a bacterial suspension from an overnight agar plate.

1.3 Example of TEM Quantification

The image below illustrates an example of a used grid square and cell that were categorized as “no visible damage” and “visible damage”, respectively.

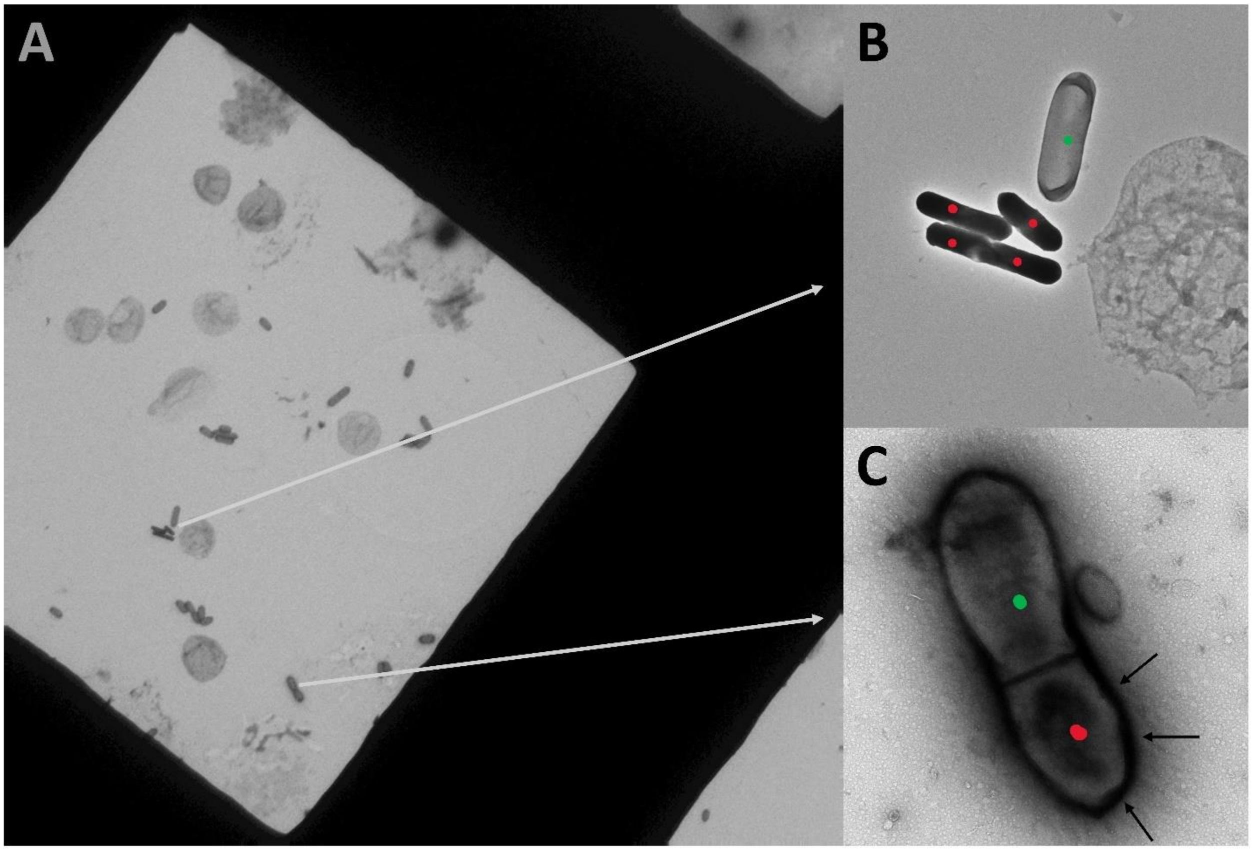

Figure S2 TEM quantification example of plectasin-treated L. monocytogenes. Figure (A) shows an example of one grid square that was used, whereas Figures (B) and (C) are magnifications of cells. Green dots on cells were categorized as "no visible damage", whereas red dots symbolized "visible damage" cells. Figure (B) shows examples of shrunk cells, and Figure (C) depicts a cell that shows cell wall depressions (highlighted by black arrows).

1.4 *AFM imaging*

The following example illustrates the process of parameter determination on *S. aureus*. To ensure the accuracy of statistical evaluation, 20 measurements were taken for each sample. The subsequent image depicts the measurement principle, wherein 0.35 x 0.35 µm squares were utilized for the assessment of specific AFM parameters.

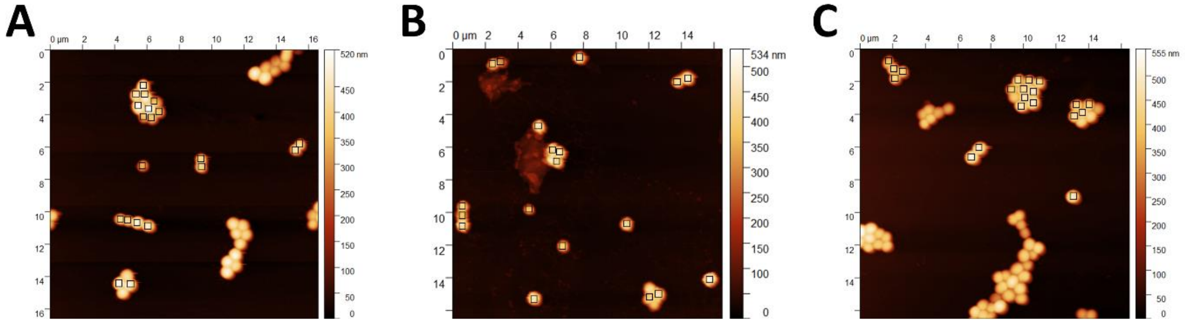

Figure S3 Illustration of AFM parameter determination using 0.35 x 0.35 µm squares for each cell. Random piles of cells were selected to determine the parameters using the square determination method. A total of 20 measurements were made on each sample to verify statistical significance.

1.5 *AFM cell parameters*

Figure S4 describes cell thickness and cell roughness parameters obtained from the atomic force microscopy measurements.

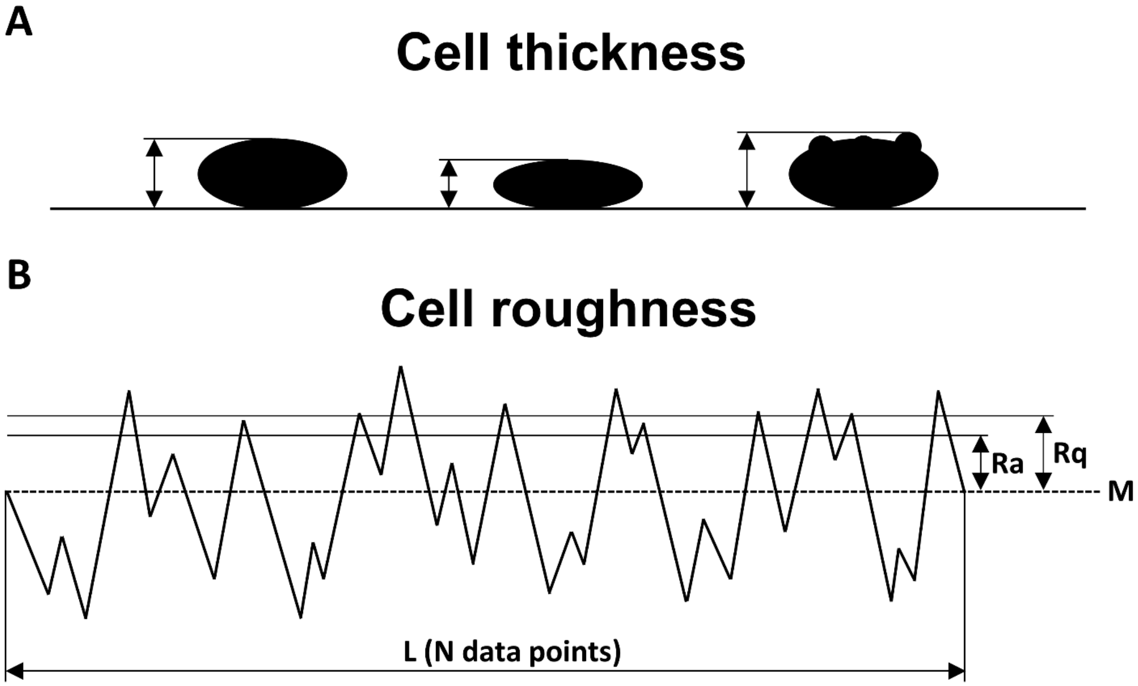

Figure S4 Schematic drawing of the parameters obtained from the atomic force microscopy measurements. (A) Representation of the measurement cell thickness. (B) Representation of cell roughness parameters. Ra, Rq, M and L represent the mean roughness, root-mean-square roughness and length of the measured cell surface area, respectively.

1.6 CASPON®-plectasin cleavage conditions

Figure S5 shows that regardless of the cleavage condition, the maximum end concentration of plectasin is limited to 1.5 g/L.

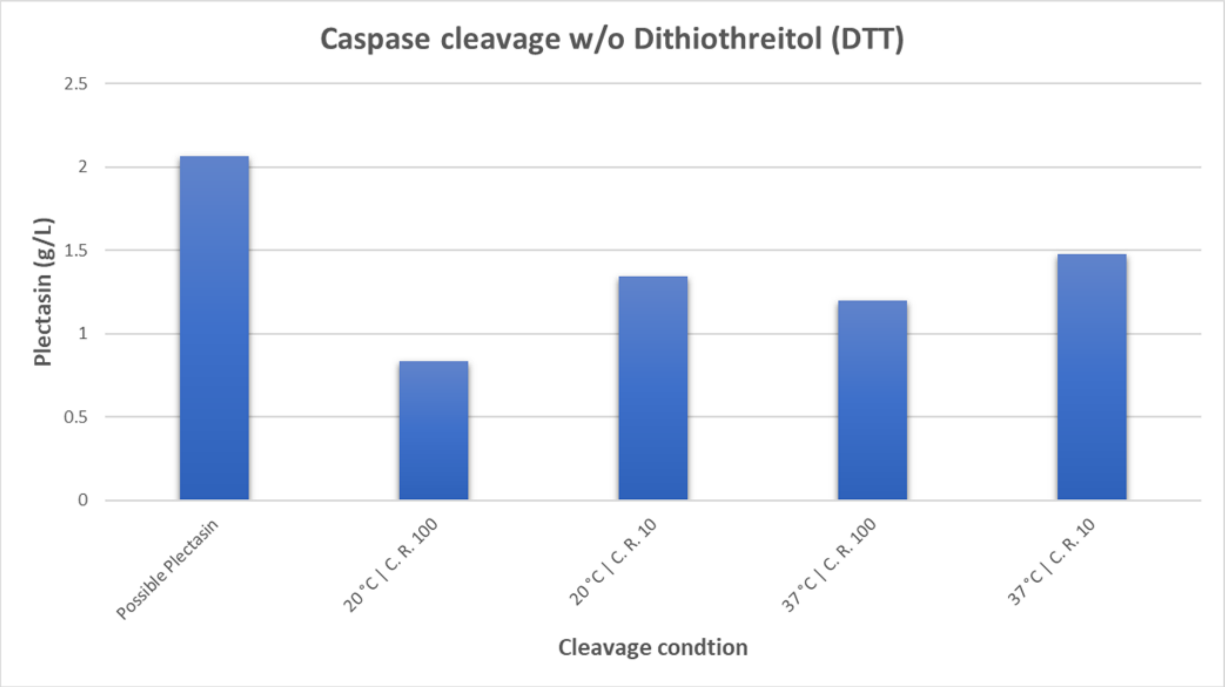

Figure S5 Cleavage experiment using CASPON®-plectasin to examine the potential cleavage of plectasin. The calculated possible plectasin was based on the used CASPON®-plectasin mass (4 g/L), assuming no losses and employing stoichiometric principles. The temperature and CASPONT® enzyme ratio (C. R.) were varied for the cleavage process.

Additional experiment (Figure S6), using dithiothreitol as a supporting agent, demonstrated that the highest achievable plectasin concentration was 1.6 g/L. This outcome is consistent with the findings of a previous study (Mygind et al. 2005), which postulated that the solubility maximum of plectasin is approximately 1.6 g/L. However, an increase in DTT beyond this concentration resulted in a reduction in the yield of plectasin.

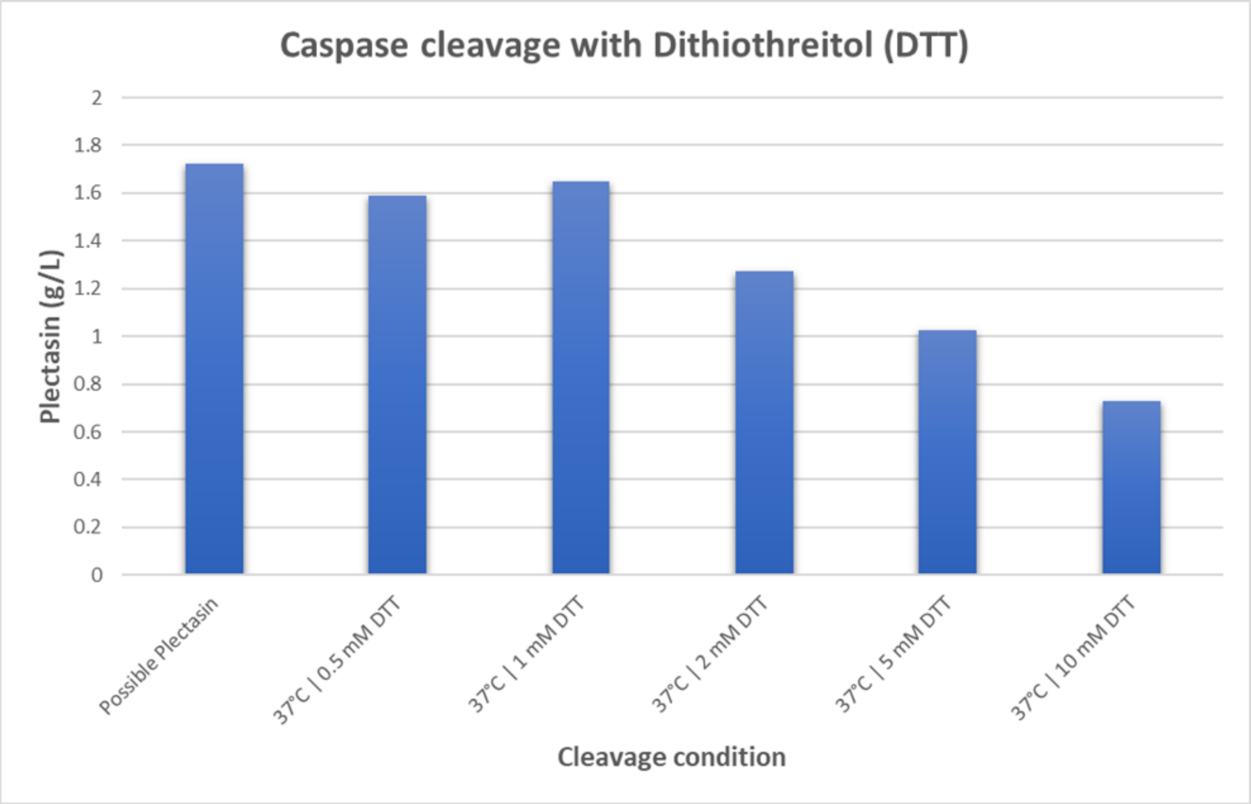

Figure S6 Cleavage experiment using CASPON®-plectasin in support with Dithiothreitol (DTT) to examine the potential cleavage of plectasin. The calculated possible plectasin was based on the used CASPON®-plectasin mass (3.4 g/L), assuming no losses and employing stoichiometric principles. The temperature of 37 °C and CASPONT® enzyme ratio of 100 were maintained while the DTT concentration was varied.

1.7 HCl purification pathway

Figure S7 summarizes all chromatographic steps of the HCl purification pathway.

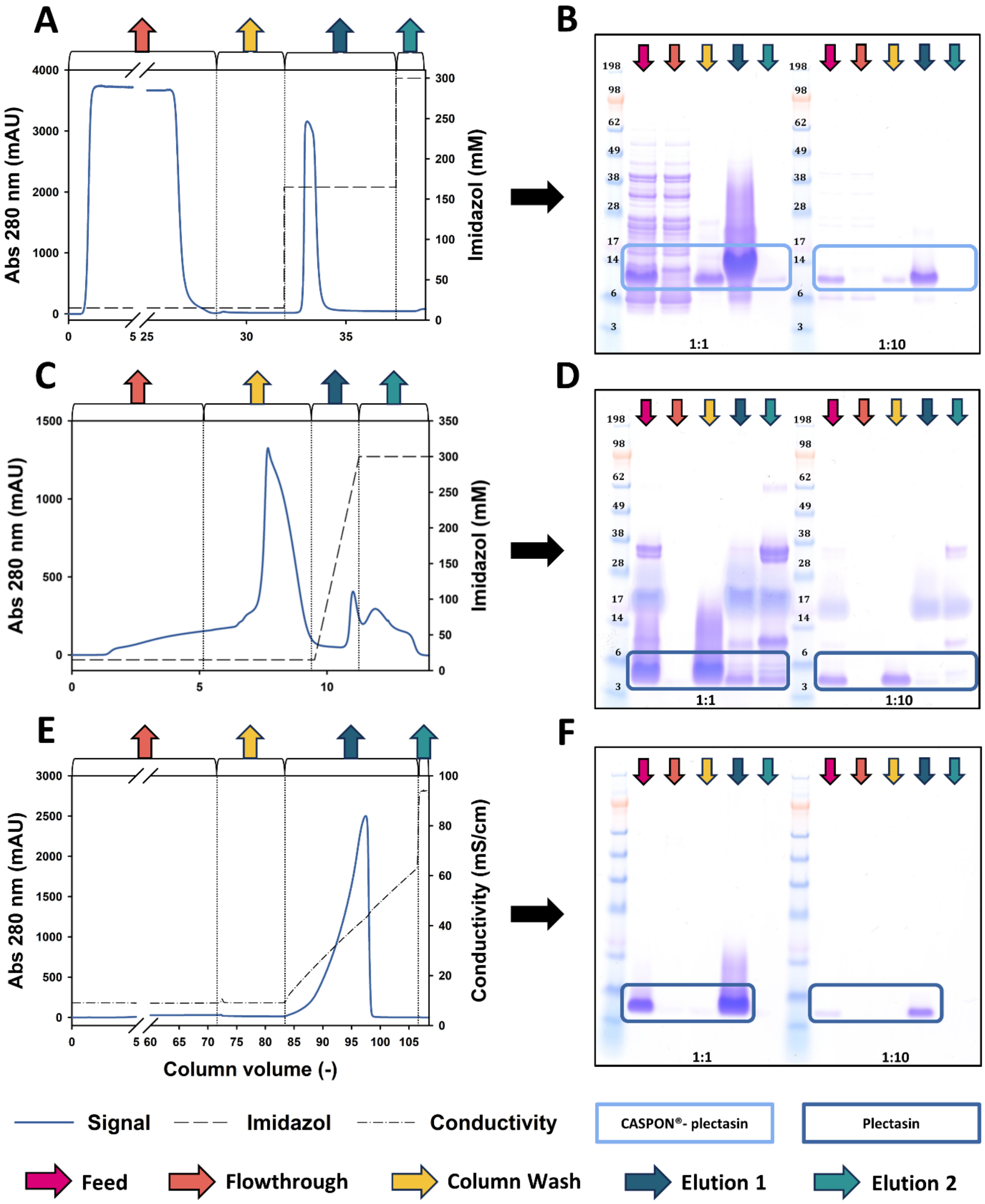

Figure S7 Chromatographic steps of the purification pathway. (A, C, E) depicts IMAC capture, subtractive IMAC and CEX, respectively. (B, D, F) show their corresponding SDS-PAGEs (Dilution 1:1 and 1:10).

1.8 Purification pathway 1:10 diluted

Figure S8 visualizes each purification step with the content of CASPON®-plectasin or plectasin with 1:10 diluted samples. It can be seen that after the cation exchange chromatography step the sample contains only pure plectasin.

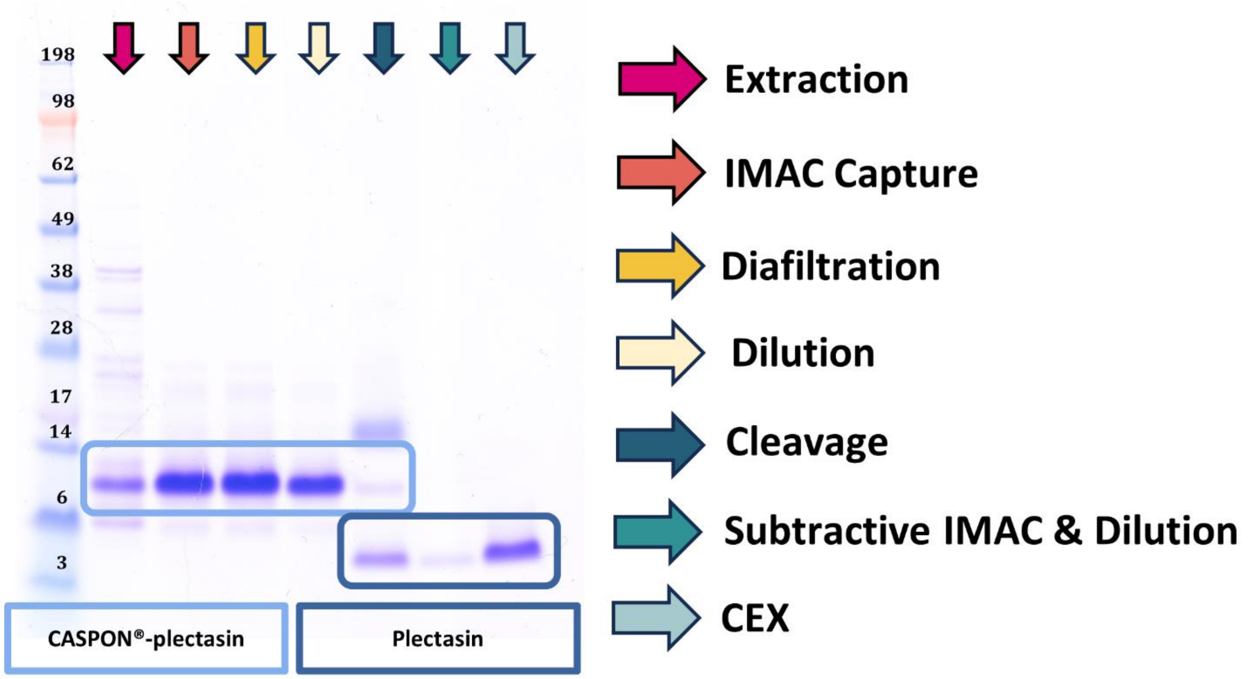

Figure S8 SDS-PAGE visualization of every purification step with 1:10 diluted samples. The peptides CASPON®-plectasin and plectasin are highlighted by light and dark blue, respectively.

1.9 Reversed phase HPLC chromatograms

Figure S9 shows reversed-phase HPLC chromatograms used to determine protein concentration and to qualitatively assess impurities.

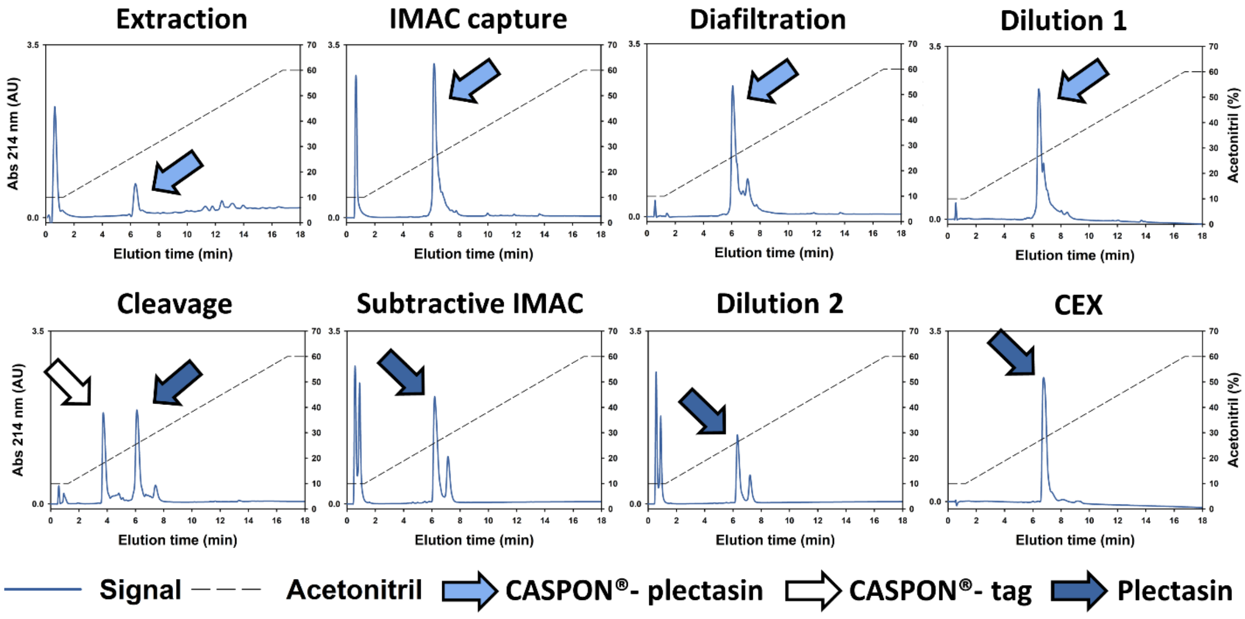

Figure S9 Reversed phase (RP)-HPLC data of the purification pathway. Each chromatogram represents the outcome of a downstream step. Blue and black dashed line represent 214 nm UV signal and imidazole gradient, respectively. Peptides are indicated by light blue (CASPON®-plectasin), white (CASPON®-tag) and dark blue (plectasin) arrow. Samples were analyzed after HCl extraction, IMAC capture, Diafiltration, dilution 1, caspase cleavage, subtractive IMAC, dilution 2, and cation exchange via RP-HPLC.

1.10 Estimated impurities

The chromatograms from the reversed-phase HPLC analysis Figure S9 were used to estimate protein impurities by dividing the product peak by the total area during elution. The estimation of peak areas was conducted using the PeakFit v4 program. Peaks between 0 and 1 min were excluded from the analysis as they are non-protein components, such as imidazole or metal ions, which are also detected at 214 nm. 214 nm chromatograms were used to detect also proteins and peptides without aromatic amino acids, such as the CASPON®-tag.

Table S2 Estimated purity of purified peptide after each purification step. The purity was calculated by analyzing the peak area of RP-HPLC chromatograms.

Purification step	Purified peptide	Purity (%)
Extraction	CASPON®-plectasin	45.1
IMAC capture	CASPON®-plectasin	85.4
Diafiltration	CASPON®-plectasin	80.4
Dilution 1	CASPON®-plectasin	78.4
Cleavage	Plectasin	41.7
Subtractive IMAC	Plectasin	72.5
Dilution 2	Plectasin	72.3
CEX	Plectasin	99.2

1.11 Mass spectrometry data

Figure S10 shows mass spectrometry data of purified CASPON®-plectasin, plectasin and reduced plectasin.

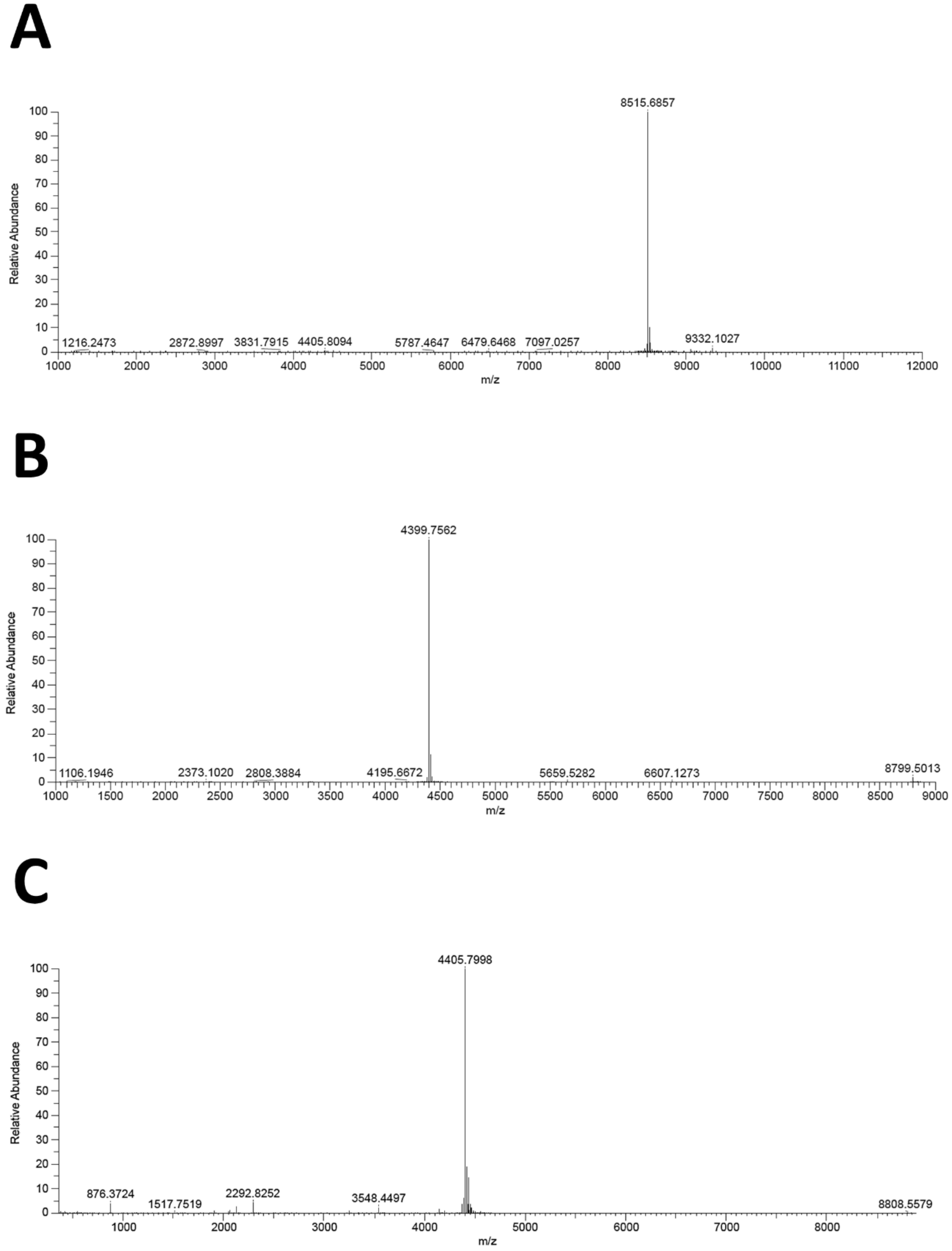

Figure S10 Mass spectrometry data of CASPON®-plectasin (A), plectasin (B) and plectasin after dithiothreitol treatment (C).

1.12 Microscopy images of treated E. coli (BL21)

Figure S11 shows microscopic images of incubated *E. coli* (BL21) cells. Untreated and CASPON®-plectasin treated bacteria form type-specific cells, whereas after high-concentration plectasin treatment, the cells greatly increased in number and differed in morphological appearance.

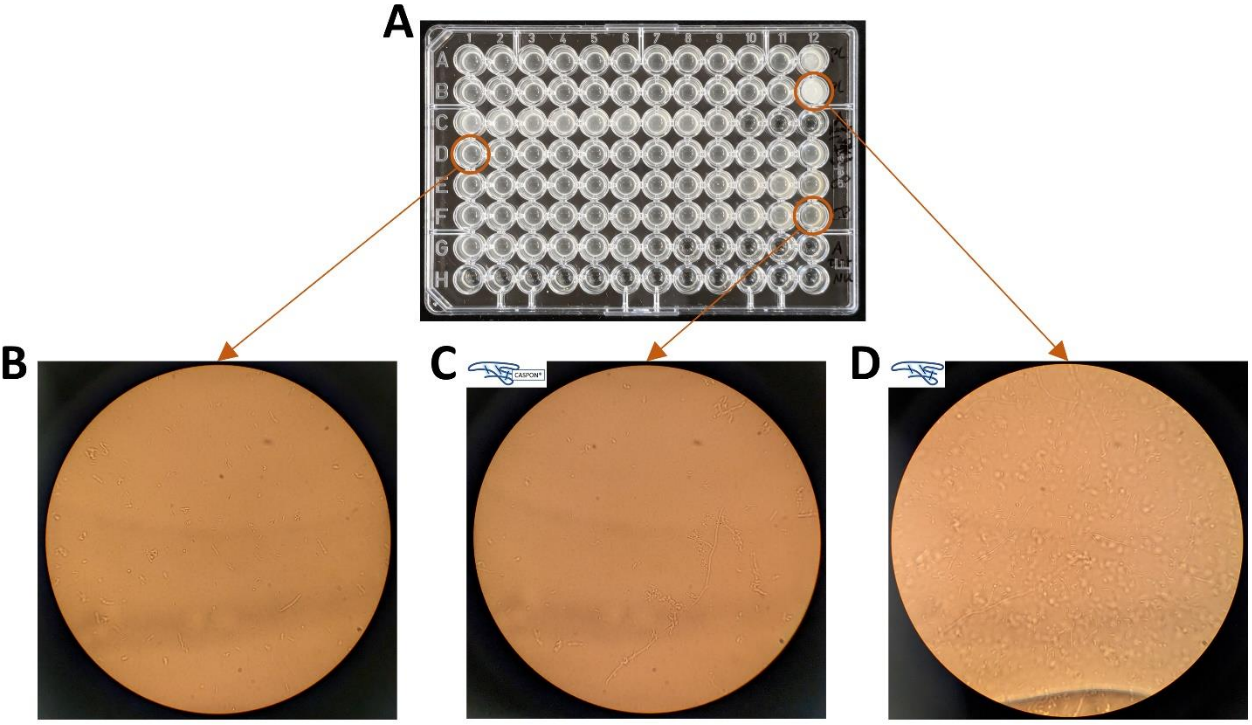

Figure S11 Microscopic visualization of untreated *E. coli* (BL21) (B), CASPON®-plectasin (16384 µg/mL) treated *E. coli* (BL21) (C) and plectasin (4096 µg/mL) treated *E. coli* (BL21) (D). (A) shows the microtiter plate of an activity assay after overnight incubation.

1.13 *Detailed TEM quantification data*

The following table presents a detailed overview of the number of cells examined per grid square used for TEM measurement.

Table S3 Detailed data set about the counted cell obtained by using TEM. The cells were categorized into two groups: “visible damage” and “no visible damage”. The mean percentage and standard deviation were calculated from these data.

			S. aureus		
	Total cells	No visible damage (n)	Visible damage (n)	No visible damage (%)	Visible damage (%)
			Untreated		
Grid Square 1	11	9	2	81.82	18.18
Grid Square 2	15	14	1	93.33	6.67
Grid Square 3	17	16	1	94.12	5.88
Grid Square 4	31	28	3	90.32	9.68
			Average	**89.90**	**10.10**
			Standard deviation	**4.88**	**4.88**
			Plectasin treated		
Grid Square 1	13	1	12	7.69	92.31
Grid Square 2	12	2	10	16.67	83.33
Grid Square 3	17	1	16	5.88	94.12
Grid Square 4	34	4	30	11.76	88.24
			Average	**10.50**	**89.50**
			Standard deviation	**4.15**	**4.15**
			CASPON®-Plectasin treated		
Grid Square 1	17	15	2	88.24	11.76
Grid Square 2	23	22	1	95.65	4.35
Grid Square 3	25	23	2	92.00	8.00
Grid Square 4	23	20	3	86.96	13.04
			Average	**90.71**	**9.29**
			Standard deviation	**3.40**	**3.40**

			E. faecalis		
	Total cells	No visible damage (n)	Visible damage (n)	No visible damage (%)	Visible damage (%)
			Untreated		
Grid Square 1	11	11	0	100.00	0.00
Grid Square 2	21	20	1	95.24	4.76
Grid Square 3	38	36	2	94.74	5.26
Grid Square 4	32	30	2	93.75	6.25
			Average	**95.93**	**4.07**
			Standard deviation	**2.41**	**2.41**
			Plectasin treated		
Grid Square 1	17	5	12	29.41	70.59
Grid Square 2	21	6	15	28.57	71.43
Grid Square 3	19	4	15	21.05	78.95
Grid Square 4	18	5	13	27.78	72.22
			Average	**26.70**	**73.30**
			Standard deviation	**3.31**	**3.31**
			CASPON®-Plectasin treated		
Grid Square 1	35	34	1	97.14	2.86
Grid Square 2	37	33	4	89.19	10.81
Grid Square 3	52	51	1	98.08	1.92
Grid Square 4	38	37	1	97.37	2.63
			Average	**95.44**	**4.56**
			Standard deviation	**3.63**	**3.63**

			S. pneumoniae		
	Total cells	No visible damage (n)	Visible damage (n)	No visible damage (%)	Visible damage (%)
			Untreated		
Grid Square 1	11	10	1	90.91	9.09
Grid Square 2	18	15	3	83.33	16.67
Grid Square 3	21	17	4	80.95	19.05
Grid Square 4	25	21	4	84.00	16.00
			Average	**84.80**	**15.20**
			Standard deviation	**3.71**	**3.71**
			Plectasin treated		
Grid Square 1	18	2	16	11.11	88.89
Grid Square 2	37	6	31	16.22	83.78
Grid Square 3	15	2	13	13.33	86.67
Grid Square 4	12	1	11	8.33	91.67
			Average	**12.25**	**87.75**
			Standard deviation	**2.90**	**2.90**
			CASPON®-Plectasin treated		
Grid Square 1	11	10	1	90.91	9.09
Grid Square 2	13	11	2	84.62	15.38
Grid Square 3	14	12	2	85.71	14.29
Grid Square 4	31	26	5	83.87	16.13
			Average	**86.28**	**13.72**
			Standard deviation	**2.75**	**2.75**

			L. monocytogenes		
	Total cells	No visible damage (n)	Visible damage (n)	No visible damage (%)	Visible damage (%)
			Untreated		
Grid Square 1	11	11	0	100.00	0.00
Grid Square 2	17	15	2	88.24	11.76
Grid Square 3	27	24	3	88.89	11.11
Grid Square 4	20	19	1	95.00	5.00
			Average	**93.03**	**6.97**
			Standard deviation	**4.81**	**4.81**
			Plectasin treated		
Grid Square 1	11	1	10	9.09	90.91
Grid Square 2	29	10	19	34.48	65.52
Grid Square 3	11	2	9	18.18	81.82
Grid Square 4	16	3	13	18.75	81.25
			Average	**20.13**	**79.87**
			Standard deviation	**9.13**	**9.13**
			CASPON®-Plectasin treated		
Grid Square 1	23	20	3	86.96	13.04
Grid Square 2	17	16	1	94.12	5.88
Grid Square 3	22	21	1	95.45	4.55
Grid Square 4	22	20	2	90.91	9.09
			Average	**91.86**	**8.14**
			Standard deviation	**3.28**	**3.28**

References

Mygind, P. H., R. L. Fischer, K. M. Schnorr, M. T. Hansen, C. P. Sönksen, S. Ludvigsen, D. Raventós, S. Buskov, B. Christensen & L. De Maria (2005) Plectasin is a peptide antibiotic with therapeutic potential from a saprophytic fungus. *Nature,* 437**,** 975-980.
